# Impact of a package of health, nutrition, psychosocial support, and WaSH interventions delivered during preconception, pregnancy, and early childhood periods on birth outcomes and on linear growth at 24 months of age: factorial, individually randomised controlled trial

**DOI:** 10.1136/bmj-2022-072046

**Published:** 2022-10-26

**Authors:** Sunita Taneja, Ranadip Chowdhury, Neeta Dhabhai, Ravi Prakash Upadhyay, Sarmila Mazumder, Sitanshi Sharma, Kiran Bhatia, Harish Chellani, Rupali Dewan, Pratima Mittal, MK Bhan, Rajiv Bahl, Nita Bhandari, WINGS Study Group, Farhana Rafiqui, Jasmine Kaur, Medha Shekhar, Anita Kate, Gunjan Aggarwal, Runa Ghosh, Ratan Shekhawat, Kunal Kishore, Navneet Mehra, Nikita Arya, Ritu Chaudhary, Anuradha Tamaria, Savita Sapra, Tivendra Kumar, Sowmya Prakash, Poornima Modi, Neelam Kaur, Neha Tyagi, Geeta Mehto, Afifa Khatun, Sayeed Ahmed, Aparna Singh, Gulafshan Ansari, Ramanjeet Kaur, Manisha Gupta, Girish Chand Pant, Ankita Dutta, Deepak More, Mukesh Kumar, Sabreen Siraj, Farah Abbasi, Heena Chaudhary, Karishma Sharma, Sonia Kuruvilla, Anjali Chandra, Sugandha Arya, Pradeep Debata, Anita Yadav, KC Aggarwal, Sujata Das, Abhinav Jain, Rahul Sachdev, Omprakash Bansal, Raghav Aggarwal

**Affiliations:** 1 Centre for Health Research and Development, Society for Applied Studies, New Delhi, India; 2 Department of Pediatrics, Vardhman Mahavir Medical College and Safdarjung Hospital, New Delhi, India; 3 Department of Obstetrics & Gynecology, Vardhman Mahavir Medical College and Safdarjung Hospital, New Delhi, India; 4 Knowledge Integration and Translational Platform (KnIT), Biotechnology Industry Research Assistance Council (BIRAC), Department of Biotechnology, Government of India, New Delhi, India; 5 Department of Maternal, Newborn, Child and Adolescent Health, World Health Organization, Geneva, Switzerland

## Abstract

**Objective:**

To determine the effect of integrated and concurrent delivery of health, nutrition, water, sanitation and hygiene (WaSH), and psychosocial care interventions during the preconception period alone, during pregnancy and early childhood, and throughout preconception, pregnancy, and early childhood on birth outcomes and linear growth at 24 months of age compared with routine care.

**Design:**

Individually randomised factorial trial.

**Setting:**

Low and middle income neighbourhoods of Delhi, India.

**Participants:**

13 500 women were randomised to receive preconception interventions (n=6722) or routine care (n=6778). 2652 and 2269 pregnant women were randomised again to receive pregnancy and early childhood interventions or routine care. The analysis of birth outcomes included 1290 live births for the preconception, pregnancy, and early childhood interventions (group A), 1276 for the preconception intervention (group B), 1093 for the pregnancy and early childhood interventions (group C), and 1093 for the control (group D). Children aged 24 months by 30 June 2021 were included in the 24 month outcome analysis (453 in group A, 439 in B, 293 in C, and 271 in D).

**Interventions:**

Health, nutrition, psychosocial care and support, and WaSH interventions were delivered during preconception, pregnancy, and early childhood periods.

**Main outcome measures:**

The primary outcomes were low birth weight, small for gestational age, preterm, and mean birth weight. At 24 months, the outcomes were mean length-for-age z scores and proportion stunted. Three prespecified comparisons were made: preconception intervention groups (A+B) versus no preconception intervention groups (C+D); pregnancy and early childhood intervention groups (A+C) versus routine care during pregnancy and early childhood (B+D) and preconception, pregnancy, and early childhood interventions groups (A) versus control group (D).

**Results:**

The proportion with low birth weight was lower in the preconception intervention groups (506/2235) than in the no preconception intervention groups (502/1889; incidence rate ratio 0.85, 98.3% confidence interval 0.75 to 0.97; absolute risk reduction −3.80%, 98.3% confidence interval −6.99% to −0.60%). The proportion with low birth weight was lower in the pregnancy intervention groups (502/2096) than in the no pregnancy intervention groups (506/2028) but the upper limit of the confidence interval crossed null effect (0.87, 0.76 to 1.01; −1.71%, −4.96% to 1.54%). There was a larger effect on proportion with low birth weight in the group that received interventions in the preconception and pregnancy periods (267/1141) compared with the control group (267/934; 0.76, 0.62 to 0.91; −5.59%, −10.32% to −0.85%). The proportion stunted at 24 months of age was substantially lower in the pregnancy and early childhood intervention groups (79/746) compared with the groups that did not receive these interventions (136/710; 0.51, 0.38 to 0.70; −8.32%, −12.31% to −4.32%), and in the group that received preconception, pregnancy, and early childhood interventions (47/453) compared with the control group (51/271; 0.49, 0.32 to 0.75; −7.98%, −14.24% to −1.71%). No effect on stunting at 24 months was observed in the preconception intervention groups (132/892) compared with the no preconception intervention groups (83/564).

**Conclusions:**

An intervention package delivered during preconception, pregnancy, and early childhood substantially reduced low birth weight and stunting at 24 months. Pregnancy and early childhood interventions alone had lower but important effects on birth outcomes and 24 month outcomes. Preconception interventions alone had an important effect on birth outcomes but not on 24 month outcomes.

**Trial registration:**

Clinical Trial Registry—India CTRI/2017/06/008908.

## Introduction

Low birth weight (LBW) and stunting (length-for-age z score<−2 standard deviations) continue to be important impediments for children to survive and thrive, and for achievement of sustainable development goals.^1^ LBW is a complex syndrome that includes preterm birth and small-for-gestational age (SGA) babies and an overlap of both.

Globally, 20.5 million (14.6%) infants are born with LBW annually, with 48% in south Asia.^2^ LBW infants have an increased risk of morbidity, developmental and behaviour problems during childhood, and cardiometabolic disease in adulthood.^3^ ^4^ ^5^ ^6^ ^7^ ^8^ Stunting is a global problem, more so in India where the prevalence in children younger than 5 years is 37.9%, which is higher than the developing country average of 8.9%.^9^ Almost all stunting occurs in the first two years, but consequences are seen throughout life.^10^ The annual reduction rate for LBW in South Asia is about half, and that for stunting about three quarters of that required to meet the 2025 global nutrition targets.^2^ ^11^

Research addressing LBW and stunting reduction has primarily focused on nutrition interventions. These studies found modest effects. The largest impact on birth weight of 0.1 standard deviation was seen with iron folic acid supplementation, and on stunting (15% reduction) through education and provision of complementary foods.^12^ ^13^ New strategies to address these multicausal problems are required. Evaluation of interventions covering multiple domains such as health, nutrition, psychosocial care, and the environment are needed to determine if there is a large, synergistic effect when these are delivered together as a package.

There is growing recognition of the importance of interventions starting before pregnancy. Studies indicate that health, nutritional, and psychosocial status before conception could be linked to LBW and stunting.^14^ ^15^ ^16^ ^17^ Interventions starting before pregnancy would cover early pregnancy, which is important because pregnancies are reported late in regions like south Asia.^16^ ^17^

This study aimed to evaluate the effect of an integrated package of health, nutrition, psychosocial support, and water, sanitation and hygiene (WaSH) interventions delivered during pregnancy and early childhood periods on birth outcomes related to LBW and on linear growth at 24 months of age. We used a factorial design to examine the impact of the package when delivered only in the preconception period, and the combined effect of implementation of the package in preconception, pregnancy, and early childhood periods. The design also enabled us to examine the effect of maternal height reflecting intergenerational adversities and genetics on the efficacy of interventions to improve birth and child outcomes.

## Methods

### Trial design

The Women and Infants Integrated Interventions for Growth Study (WINGS) is an individually randomised trial with factorial design conducted in low income to lower middle income neighbourhoods of Delhi, India. The study methods have been previously published.^18^

### Participants

Married women aged 18-30 years with no or one child were identified through a survey. Women living in temporary housing and those moving away were excluded. Written informed consent was obtained for participation. Enrolled women were randomised to receive preconception interventions or routine care (first randomisation). Women were followed up until pregnant or up to 18 months after enrolment. Women received ultrasonographic confirmation of pregnancy and were then randomised (second randomisation) to pregnancy and early childhood interventions or to routine care. Outcomes were measured at birth and 24 months of age.

### Randomisation and masking

Women were randomised using permuted blocks and stratified by maternal height (<150 cm and ≥150 cm). Group allocation (1:1) was through a web based system. The two step randomisation resulted in four groups: preconception, pregnancy and early childhood interventions (A), preconception interventions only (B), pregnancy and early childhood interventions only (C), no preconception interventions, and routine pregnancy and early childhood care (D). The randomisation list was prepared by an independent statistician.

We could not mask participants and teams because of the nature of the interventions. Outcomes were assessed by an independent team not involved in delivering interventions or aware of the group allocation before measurements.

### Study interventions

The interventions were in four domains: health, nutrition, psychosocial care and support, and WaSH during the preconception, pregnancy, and early childhood (0-24 months) periods (table 1). These interventions were selected after a review of systematic reviews based on evidence of their impact on any of the following measures: preterm birth, SGA, LBW, birth weight, birth length, stunting at 24 months, and length-for-age z score at 24 months. The available evidence was discussed and selection of interventions was finalised in consultation with the technical advisory group for the study.

**Table 1:** Summary of the intervention package

Period and health	Nutrition	Psychosocial support	WaSH
**Preconception**			
Screen and treat medical conditions	Provide IFA and MMN; provide egg or milk if body mass index <21; screen and treat malnutrition, anaemia	Promote positive thinking and problem solving skills	Promote personal, menstrual and hand hygiene
**Pregnancy**			
At least eight antenatal contacts, screen and treat GHT or preeclampsia, GDM, hypothyroidism, UTI, RTI, calcium supplement	Provide IFA and MMN, locally prepared snacks (210 and 400 kcal, 2 and 21 g protein in second and third trimester, respectively), provide milk (180 ml daily), provide one additional hot cooked meal (500 kcal, 20 g protein) if body mass index <18.5 or inadequate gestational weight gain	Promote positive thinking and problem solving skills	Provide water filters, soap, hand washing station, disinfectant
**Early childhood**			
Empower family to identify danger signs and seek care early	0-6 months: lactation support for early and exclusive breastfeeding; 6-24 months: promote timely CF and continued breastfeeding; provide supplementary food (125 and 250 kcal, 2.5 and 5 g protein in months 6-11 and 12-23, respectively); double supplement if inadequate weight gain	Promote early child play and responsive care	Provide play mat and potty
**Postnatal 6 months: mother**			
Arrange postnatal visit at six weeks	Provide IFA, MMN, calcium, and vitamin D; locally prepared snacks (600 kcal, 20 g protein); provide milk (180 mL daily)	Promote positive thinking and problem solving skills	Same as in pregnancy

Control group: weekly iron folic acid supplementation as part of the national programme during preconception period; routine antenatal and postnatal care for the mother and routine newborn and infant care during early childhood. CF=complementary feeding, GDM: gestational diabetes mellitus, GHT: gestational hypertension, IFA: iron folic acid, MMN: multiple micronutrients; RTI=reproductive tract infection; UTI=urinary tract infection; WaSH=water, sanitation and hygiene.

### Procedures

#### Preconception period

Women in the control group were advised to seek care from government sources (free of cost) to access family planning services and weekly iron folic acid supplementation (table S1, supplementary appendix). The study team screened women in the intervention group for reproductive tract infections, tuberculosis, thyroid disorders, hypertension, prediabetes, diabetes, undernutrition, anaemia, and depressive symptoms and treated them according to standard protocols.^18^ Contraceptives were provided to women who had started living with their husbands in the past 12 months, had a child aged 12 months or younger, or had severe undernutrition, moderate to severe anaemia, hypothyroidism, reproductive tract infection, diabetes, or hypertension. We gave these women a multiple micronutrient tablet to be taken three times a week. Weekly tablets of iron folic acid were provided to women with no anaemia. Women with a body mass index <21 were given one egg or milk (70 kcal, 6 g protein) daily for six days a week. Locally prepared snacks were also given to undernourished women (body mass index <16: 1000 kcal/day and 20-22 g protein/day; body mass index 16-18.40: 500 kcal/day and 12-14 g protein/day). We gave women an albendazole tablet twice a year. Women also received counselling on adequate diets, on positive thinking and problem solving skills using an adaptation of the thinking health module of the World Health Organization,^19^ and on menstrual and hand hygiene (table S2, supplementary appendix).

Trial community workers or Sanginis (friends) visited homes weekly to reinforce interventions, replenish supplies, and help to manage health conditions. Women with moderate to severe anaemia, thyroid disorders, reproductive tract infections, or undernutrition were contacted more frequently.

#### Pregnancy identification

Women randomised to the preconception intervention and the no preconception intervention groups were contacted monthly to enquire about missed periods. Women had a transabdominal ultrasound to confirm pregnancy when two consecutive missed periods were reported.

### Pregnancy and early childhood periods

#### Pregnancy

Women in the control group were advised to register for antenatal care at a government or private facility, have at least four antenatal care check-ups, consume iron folic acid, calcium, vitamin D daily throughout pregnancy, access supplementary foods through the Integrated Child Development Services (ICDS) scheme and plan to deliver in health facilities (table S1, supplementary appendix). In the intervention group, pregnant women were screened for anaemia, gestational diabetes mellitus, thyroid disorders, gestational hypertension, gestational weight gain, asymptomatic bacteriuria, reproductive tract infections, and depressive symptoms during monthly antenatal care visits. Hospital registration for childbirth was encouraged. Women were given micronutrient supplements, iron folic acid, calcium and vitamin D daily, and albendazole once during pregnancy. Weekly supplies of locally prepared snacks (210 kcal, 2 g protein in the second trimester, and 400 kcal, 21 g protein in the third trimester) were provided for daily consumption in women with body mass index <25. Milk was provided six days a week to all women and its consumption was observed. Weight of pregnant women was monitored every month and inadequate weight gain was identified according to Institute of Medicine guidelines.^20^ Women with inadequate weight gain were provided nutritional counselling and a hot cooked meal six days a week (500 kcal, 20 g protein) until delivery. We screened and treated these women for infections (urinary tract infection, reproductive tract infection, dental infection, tuberculosis). Positive thinking and problem solving skills were promoted.^19^ Water filters and handwashing stations were installed, and water storage bottles, soap, and disinfectants provided to families (table S2, supplementary appendix).

#### After delivery: mother

Women in the control group were advised to go for a postnatal health check-up, and to consume iron folic acid, calcium, vitamin D, and supplementary foods daily through the ICDS scheme (table S1, supplementary appendix). In the intervention group, trial community workers, Prernas (inspiration), enabled postnatal visits to facilities. Snacks, milk (600 kcal, 20 g protein), micronutrient supplements, iron folic acid, calcium, and vitamin D were given for six months to meet additional requirements during lactation. Counselling on positive thinking and problem solving skills, screening and management of depressive symptoms, and WaSH interventions were continued. Hand washing, use of diapers, appropriate disposal of faeces, and a clean play area for the child were promoted (table S2, supplementary appendix).^18^

#### Early childhood

In the control group, mothers were advised to breastfeed their babies exclusively for the first six months, and continue breastfeeding for at least two years. They were also encouraged to arrange home visits by the community health workers in the first 42 days of life, and to collect supplementary food from ICDS and iron folic acid from 6 to 24 months (table S1, supplementary appendix).

In the intervention group, newborns were home visited within 24 hours of birth or hospital discharge, five times in the first month, monthly until 12 months, and three monthly thereafter up to 24 months of age.^18^ Additional visits were made for babies born preterm, LBW, and for mothers with breastfeeding problems. Exclusive breastfeeding was promoted until six months. Mothers were trained to feed expressed breastmilk to babies born preterm. Lactation counsellors supported mothers with breastfeeding problems. In the first six months of life, vitamin D (400 IU) was provided daily to all infants.^21^ Iron supplementation was given from two weeks for the very LBW and from six weeks to LBW infants until six months of age.^22^ Complementary food supplements (milk-cereal mix) were started from six months of age and continued to 24 months of age. From 6 to 12 months of age, 125 kcal and 2.5 g protein were given; the amount was doubled from 12 to 24 months of age (250 kcal and 5 g protein). The food supplement provided 40-60% of daily energy requirements and contained 80-100% of the required daily amounts of micronutrients. Additionally, nutrient dense recipes made with locally available foods were provided to families and responsive feeding was promoted. An iron supplement was also given.

Weights were measured during home visits and children with inadequate weight gain (<15th centile from birth to 6 months and <25th centile weight velocity/month from 6 to 24 months) were referred to lactation counsellors and paediatricians.^23^ Mothers were offered additional packets of food supplements until infants no longer showed inadequate weight gain. The increased amount was given as additional packets of milk-cereal mix or other locally available foods based on the mother’s preference.

Mothers were taught age specific child play, responsive care, and stimulation activities. Development milestones were assessed three monthly by Prernas.^24^ Compliance to interventions was assessed by study workers through observation or by asking mothers during home visits (table S2, supplementary appendix).

### Outcomes

The primary outcomes at birth were proportion LBW (birth weight <2500 g), preterm birth (ultrasound confirmed gestational age at birth <37 completed weeks), SGA (birth weight centile <10th using INTERGROWTH-21st standards), and mean birth weight and length. At 24 months of age, these were mean length-for-age z scores and proportion stunted. The key secondary outcomes for women were infection, hypothyroid status, weight, anaemia status, and depressive symptoms at the end of preconception, during pregnancy, and postpartum. The key secondary outcomes for children were proportion stunted (length-for-*age z score <*–2 standard deviations of the WHO child growth standards), wasted (weight-for-height z score <−2 standard deviations of the WHO child growth standards), underweight (weight-for-age z score <−2 standard deviations of the WHO child growth standards) during 6-24 months, weight and length trajectories from birth to 24 months, anaemia status at 24 months, morbidity, and hospital admission from birth to 24 months (table S3, supplementary appendix).

Outcomes were assessed by an independent team at the end of the preconception period, after 26-28 and 35-37 weeks of gestation, within the first week of birth, at one month, and three monthly thereafter until infant age 24 months. The infant’s weight, length, mid-upper arm circumference, and head circumference were measured by a pair of workers independently and repeated if the difference was outside the prespecified limit. The two readings were averaged and used for analysis. Ten per cent of measurements were repeated independently. The serious adverse events for this study were severe allergic reactions to supplements and death; these were reported to the data safety monitoring committee (DSMC) and the ethics committee.^18^

### Statistical analysis

We developed an a priori analysis approach for this factorial design, randomised controlled trial to evaluate the three study hypotheses by making three comparisons for all primary and secondary outcomes: effect of preconception interventions (groups A+B *v* C+D); effect of pregnancy and early childhood interventions (groups A+C *v* B+D); and the combined effect of interventions from preconception until two years after birth (group A *v* D). We also displayed comparisons of individual groups and assessed interaction between preconception interventions and pregnancy and early childhood interventions for all primary outcomes. We used 98.3% confidence intervals of effect sizes for all primary and secondary outcomes to adjust for the three comparisons (significance level 0.05/3 or 0.017).

The study had multiple primary outcomes related to LBW (proportion LBW, SGA, and preterm, and mean birth weight and length) and stunting (proportion stunted and mean length-for-age z score at 24 months of age). Our a priori decision was not to adjust for multiple comparisons because the primary outcomes were likely to be correlated, we were not addressing a universal null hypothesis, and formal adjustments for multiplicity are unlikely to enhance interpretation.^25^ However, for those who strongly believe in adjustment for multiple primary endpoints, we conducted a post hoc sensitivity analysis where we adjusted P values for multiple primary outcomes using the Holm-Bonferroni method.^26^ In this sensitivity analysis, we adjusted for a total of 21 comparisons; seven primary outcomes (five at birth and two at 24 months) and three, two group comparisons (table S4, supplementary appendix).

Sample sizes were calculated for 90% power (80% for preterm birth) and 95% confidence interval for comparison between groups. We assumed at least 0.15 standard deviation mean difference for birth weight or length and length-for-age z score at 24 months, 25% relative reduction for LBW, preterm birth and SGA, and stunting at 24 months for the impact of preconception (groups A+B *v* C+D) or pregnancy and early childhood interventions (groups A+C *v* B+D). We used 0.20 standard deviation mean difference for birth weight or length, length-for-age z score at 24 months, 30% relative reduction for LBW, preterm birth, SGA, and stunting at 24 months for the combined effect of preconception and pregnancy and early childhood interventions compared with the control group (group A *v* D). We aimed for 1100 live births (based on the outcome with the largest sample size) and 600 children at 24 months in each of the four groups. To achieve 4400 live births, we enrolled 13 500 women in the preconception period.^18^

The DSMC conducted interim analyses when 50% of the sample sizes for primary outcomes at birth and at 24 months were achieved (March 2020 and May 2021). After the interim analysis for 24 month outcomes, the DSMC recommended stopping the study based on indisputable evidence of beneficial effects using the prespecified stopping rule at P<0.001 for outcomes at 24 months.^27^ Data collection for primary outcomes ended on 30 June 2021. We assessed similarity of means or proportions of baseline characteristics across the groups to check whether randomisation was successful. Intention-to-treat analysis was conducted.

We used generalised linear models of the Poisson family with a log link function and Gaussian family with an identity link function to calculate incidence rate ratio and mean difference for binary and continuous outcomes, respectively. We also calculated absolute risk reduction with 98.3% confidence interval for primary and secondary outcomes at birth and 24 months. The final models were adjusted for place of birth, family possessing a below poverty line card, women’s height, and women’s body mass index which were potential confounders. We also adjusted the analysis for clustering due to twins. 

The intervention effect on secondary outcomes was assessed using the same models as for primary outcomes. We used Kernel weighted local polynomial smoothing technique to create length-for-age z score from birth to 24 months for all three comparisons. The relative measures of effect on key primary outcomes within each of the prespecified subgroup analyses were estimated and presented as forest plots. Data analysis was conducted with Stata version 16.0.

### Patient and public involvement

We have been working in the study setting for over two decades and understand the study population well. Reduction in the proportion of LBW babies and better care during pregnancy are key priorities. We conducted formative research for a year to understand the needs and aspirations of the families, to assess their behaviour and perceptions about the interventions in different domains, and to determine the best way of delivering interventions at home. The interventions were finalised in consultation with the Study Technical Advisory Group which included representatives from the community who were familiar with the issues. We plan to organise dissemination meetings to share the findings with the community and policymakers.

## Results

**Fig 1:**
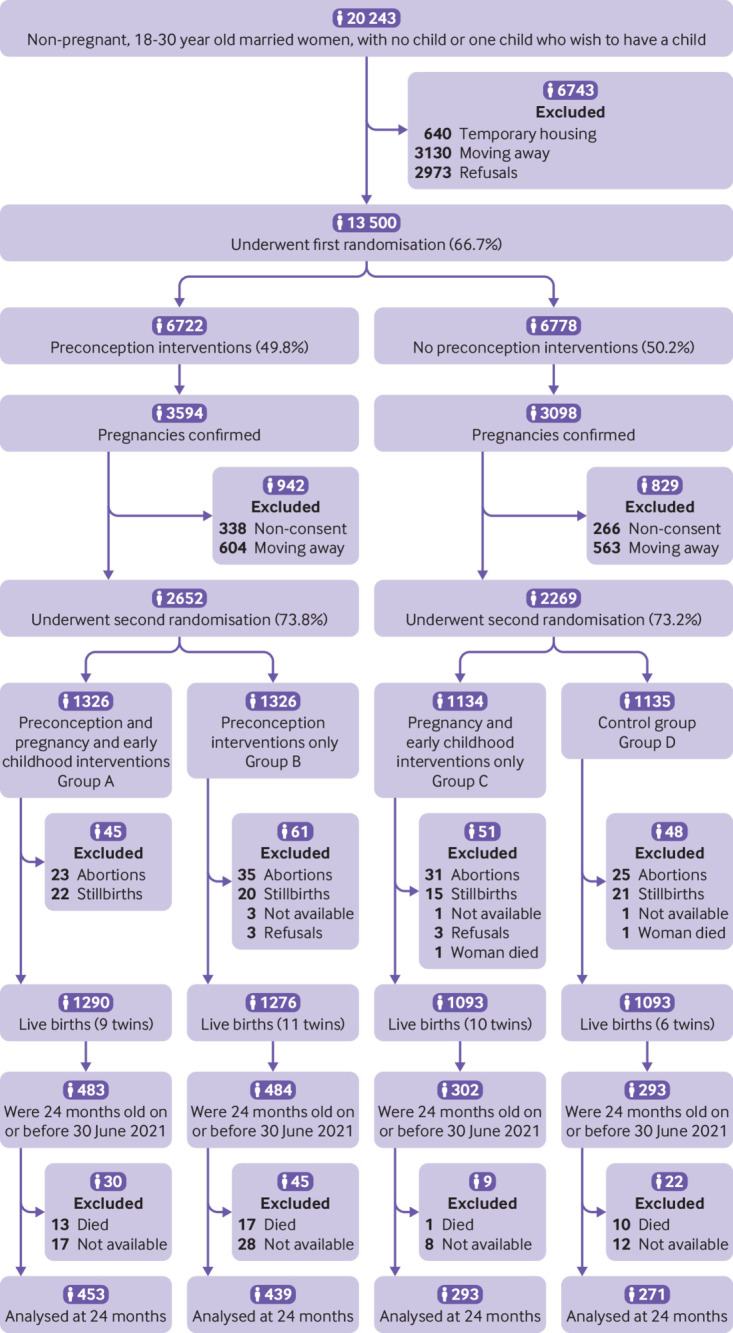
Screening, enrolment, randomisation, and follow-up

Of the 20 243 women screened between 1 July 2017 and 30 December 2019, 13 500 underwent first randomisation to receive preconception interventions (n=6722) or routine care (n=6778). Of the women randomised in the preconception intervention group, 2938 (43.7%) did not get pregnant during the 18 month follow-up period, 3594 reported pregnancies, and 190 were censored prematurely. Similarly, in the routine care group, 3562 (52.6%) women did not get pregnant during the follow-up period, 3098 reported pregnancy, and 118 were censored prematurely. Women with an ultrasound confirmed pregnancy—2652 (73.8%) in the preconception and 2269 (73.2%) in the routine care group—underwent a second randomisation to receive pregnancy and early childhood interventions or routine care. The number of live births was 1290 in group A (preconception and pregnancy and early childhood interventions), 1276 in group B (preconception interventions), 1093 in group C (pregnancy and early childhood interventions), and 1093 in group D (control). Children who reached 24 months of age by 30 June 2021 (DSMC decision) were included in the analysis for 24 month outcomes: 453 in group A, 439 in group B, 293 in group C, and 271 in group D (fig 1).

The baseline characteristics of women at both randomisations were similar except for women’s height, proportion underweight, families possessing a below poverty line card, and place of birth (table 2). The median gestational age when pregnancy interventions were started was 10.6 weeks (interquartile range 9.9–12.3 weeks).

**Table 2:** Baseline characteristics of enrolled women at first and second randomisation

Characteristics at enrolment	First randomisation			Second randomisation			
	Preconception interventions (n=6722)	No preconception interventions (n=6778)		Preconception, pregnancy, and early childhood interventions (group A, n=1326)	Preconception interventions only (group B, n=1326)	Pregnancy and early childhood interventions only (group C, n=1134)	Control (group D, n=1135)
Age of women (years), mean (SD)	24.2 (3.1)	24.2 (3.1)		24.5 (3.1)	24.5 (3.0)	24.5 (3.1)	24.6 (3.1)
Height of women (cm), mean (SD)	152.2 (5.7)	152.2 (5.6)		152.1 (5.7)	152.1 (5.6)	152.6 (5.6)	152.1 (5.6)
Height <150 (cm)	2340 (34.8)	2357 (34.8)		465 (35.1)	463 (34.9)	381 (33.6)	384 (33.8)
Body mass index							
≥25	1798 (26.8)	1813 (26.8)		321 (24.2)	345 (26.0)	274 (24.2)	295 (26.0)
18.5-24.99	3774 (56.1)	3841 (56.7)		811 (61.2)	806 (60.8)	687 (60.6)	652 (57.4)
<18.5	1150 (17.1)	1124 (16.6)		194 (14.6)	175 (13.2)	173 (15.3)	188 (16.6)
Joint or extended family*	3922 (58.6)†	3907 (57.6)		884 (66.7)	861 (65.0)	752 (66.3)	717 (63.2)
Women schooling ≥12 years	3286 (49.1)†	3444 (50.8)		664 (50.1)	648 (48.9)	586 (51.7)	575 (50.7)
Homemaker	6328 (94.6)†	6421 (94.7)		1260 (95.0)	1254 (94.6)	1082 (95.4)	1097 (96.7)
Family has below poverty line card	129 (1.9)†	357 (5.3)		27 (2.0)	30 (2.3)	57 (5.0)	72 (6.3)
Family covered by health insurance scheme	555 (8.3)†	975 (14.4)		90 (6.8)	122 (9.2)	171 (15.1)	169 (14.9)
Place of birth‡							
Large hospital	—	—		923 (69.6)	442 (33.3)	762 (67.2)	402 (35.4)
Small hospital or birthing centre	—	—		335 (25.3)	729 (55.0)	293 (25.8)	611 (53.8)
Home birth	—	—		45 (3.4)	116 (8.8)	44 (3.9)	95 (8.4)

Data are numbers (%) unless indicated otherwise.

SD=standard deviation.

* Joint or extended family: adult relatives other than enrolled woman’s husband and children living together in household.

† Information missing for 32 participants.

‡ Information missing for 23 participants in group A, 39 in group B, 35 in group C, and 27 in group D because they had an abortion.

Compliance with interventions was high. Around 90% of women were screened and received treatment for different morbidities during the preconception and pregnancy periods. Women and children consumed nutritional supplements for around 75% of the follow-up period (tables S5-S9, supplementary appendix). Around 97% of women received counselling on positive thinking and problem solving skills, and 98% of children on early child play and responsive care.

### Maternal outcomes

At the time of the second randomisation after confirmation of pregnancy, the proportion of women with reproductive tract infections was lower (incidence rate ratio 0.68, 98.3% confidence interval 0.59 to 0.80; absolute risk reduction −7.49%, 98.3% confidence interval −10.57% to −4.41%), and mean haemoglobin was 0.56 g/dL higher (98.3% confidence interval 0.48 to 0.64 g/dL) in the preconception intervention groups than in the groups that did not receive these interventions. The proportion of women with hypothyroidism (0.83, 0.68 to 1.03; −1.96%, −4.34% to 0.42%) and body mass index <18.5 (0.88, 0.73 to 1.05; −1.98%, −4.64% to 0.68%) was lower in groups that received preconception interventions than in groups that did not receive preconception interventions, but the upper limit of the confidence interval crossed null effect (table 3).

**Table 3:** Secondary outcomes in women at time of second randomisation

	Preconception interventions	No preconception interventions	Incidence rate ratio or mean difference (98.3% CI)	Absolute risk reduction (%)‡ (98.3% CI)
Reproductive tract infections, n/total (%)*	427/2648 (16.1)	533/2265 (23.5)	0.68 (0.59 to 0.80)	−7.49 (−10.57 to −4.41)
Hypothyroidism, n/total (%)*	255/2485 (10.3)	259/2110 (12.3)	0.83 (0.68 to 1.03)	−1.96 (−4.34 to 0.42)
Prediabetes or diabetes, n/total (%)*	59/2139 (2.8)	62/1896 (3.3)	0.81 (0.52 to 1.25)	−0.35 (−1.62 to 0.93)
Hypertension, n/total (%)*	51/1762 (2.9)	45/1674 (2.7)	1.04 (0.64 to 1.70)	0.07 (−1.29 to 1.43)
Anaemia status	n=2560	n=2147	—	—
Haemoglobin levels (g/dL), mean (SD)†	11.9 (1.0)	11.4 (1.4)	0.56 (0.48 to 0.64)	—
No anaemia*	1302 (50.9)	763 (35.5)	1.43 (1.28 to 1.60)	15.93 (11.39 to 20.47)
Mild anaemia*	878 (34.3)	691 (32.2)	1.06 (0.94 to 1.20)	1.96 (−2.08 to 5.99)
Moderate anaemia*	375 (14.7)	645 (30.0)	0.49 (0.42 to 0.57)	−15.14 (−18.50 to −11.78)
Severe anaemia*	5 (0.2)	48 (2.2)	0.09 (0.03 to 0.28)	−2.00 (−2.79 to −1.21)
Body mass index	n=2652	n=2269	—	—
≥25*	666 (25.1)	569 (25.1)	1.00 (0.87 to 1.15)	0.05 (−3.39 to 3.50)
18.5-24.99*	1617 (61.0)	1339 (59.0)	1.03 (0.95 to 1.13)	1.93 (−3.37 to 7.23)
<18.5*	369 (13.9)	361 (15.9)	0.88 (0.73 to 1.05)	−1.98 (−4.64 to 0.68)
Birth interval				
Months between previous birth and pregnancy	n=1500	n=1325	—	—
Mean (SD)†	38.8 (23.1)	38.6 (20.9)	0.11 (−1.84 to 2.05)	—
Median (IQR)	33.4 (22.2-50.3)	34.8 (23.0-49.2)	—	—
Months between marriage and pregnancy	n=1147	n=943	—	—
Mean (SD)†	20.4 (18.0)	20.6 (18.0)	−0.41 (−2.29 to 1.47)	—
Median (IQR)	15.3 (7.9-26.6)	15.6 (8.3-26.8)	—	—
PHQ-9 score ≥10, n/total (%)*	60/2648 (2.3)	55/2255 (2.4)	0.92 (0.59 to 1.43)	−0.27 (−1.35 to 0.82)

Data are numbers (%) unless indicated otherwise. Reproductive tract infection based on symptoms: any symptoms of vaginal discharge, itching, burning, swelling, ulcer in genital region, swelling and pain lower abdomen; hypothyroidism: thyroid stimulating hormone ≥4.0 mIU/mL; prediabetes or diabetes: HbA_1c_>5.7%; hypertension: systolic blood pressure ≥140 mm Hg and diastolic blood pressure ≥90 mm Hg; no anaemia: haemoglobin (Hb)≥12g/dL; mild anaemia: Hb 10-11.99 g/dL; moderate anaemia: Hb 8-9.99 g/dL; severe anaemia: Hb<8g/dL.

CI=confidence interval; IQR=interquartile range; PHQ-9=patient health questionnaire; SD=standard deviation.

* Incidence rate ratio (98.3% CI), †mean difference (98.3% CI), adjusted for family possesses below poverty line card, woman’s height, woman’s body mass index; not corrected for multiple outcomes or comparisons. ‡Absolute risk reduction (98.3% CI), adjusted for family possesses below poverty line card, woman’s height, woman’s body mass index.

At 35-37 weeks of gestation, haemoglobin concentration (mean difference 0.68 g/dL, 98.3% confidence interval 0.56 to 0.80 g/dL) was higher, and the proportion with moderate anaemia (incidence rate ratio 0.36, 98.3% confidence interval 0.28 to 0.47; absolute risk reduction −13.45%, 98.3% confidence interval −16.42% to −10.48%), severe anaemia (0.03, 0 to 0.31; −1.69%, −2.46% to −0.92%), reproductive tract infection (0.66, 0.56 to 0.79; −8.33%, −11.88% to −4.78%), preeclampsia or eclampsia (0.55, 0.33 to 0.91; −1.68%, −3.12% to −0.24%) was lower. Gestational weight gain between enrolment and 35 weeks of gestation was 1.42 kg (98.3% confidence interval 1.15 to 1.70) higher in the pregnancy intervention groups than in the no pregnancy intervention groups (A+C *v* B+D; table 4).

**Table 4:** Secondary outcomes in women at 35-37 weeks of pregnancy

Outcomes	Group A	Group B	Group C	Group D	Preconception intervention *v* no preconception intervention (A+B *v* C+D)			Pregnancy intervention *v* no pregnancy intervention (A+C *v* B+D)			Preconception and pregnancy intervention *v* no preconception and pregnancy intervention (A *v* D)		
					IRR (98.3% CI)	ARR¶ (%) or MD (98.3% CI)		IRR (98.3% CI)	ARR¶ (%) or MD (98.3% CI)		IRR (98.3% CI)	ARR¶ (%) or MD (98.3% CI)	
RTI based on symptoms, n/total (%)*	193/1150 (16.8)	251/1008 (24.9)	175/964 (18.2)	230/855 (26.9)	0.93 (0.79 to 1.10)	−1.44 (−4.97 to 2.10)		0.66 (0.56 to 0.79)	−8.33 (−11.88 to −4.78)		0.62 (0.48 to 0.79)	−9.76 (−14.91 to −4.61)	
Hypertension, n/total (%)*	57/941 (6.1)	71/829 (8.6)	55/839 (6.6)	61/741 (8.2)	0.94 (0.69 to 1.28)	0.18 (−2.00 to 2.36)		0.81 (0.58 to 1.13)	−1.86 (−4.05 to 0.33)		0.71 (0.44 to 1.14)	−1.70 (−4.88 to 1.48)	
Anaemia status (n=1073, 932, 888, 788 for groups A-D, respectively)													
Haemoglobin (g/dL), mean (SD)†	11.7 (1.3)	11.2 (1.5)	11.8 (1.2)	10.9 (1.7)	—	0.08 (−0.04 to 0.19)		—	0.68 (0.56 to 0.80)		—	0.77 (0.59 to 0.95)	
No anaemia*	815 (76.0)	533 (57.2)	694 (78.2)	430 (54.6)	1.01 (0.91 to 1.11)	0.43 (−6.08 to 6.95)		1.35 (1.22 to 1.51)	21.00 (14.60 to 27.39)		1.38 (1.18 to 1.61)	21.84 (12.85 to 30.83)	
Mild anaemia*	174 (16.2)	217 (23.3)	140 (15.8)	158 (20.1)	1.08 (0.90 to 1.30)	1.53 (−1.87 to 4.93)		0.74 (0.61 to 0.91)	−5.67 (−9.10 to −2.23)		0.82 (0.61 to 1.08)	−3.67 (−8.49 to 1.15)	
Moderate anaemia*	84 (7.8)	175 (18.8)	53 (6.0)	177 (22.5)	0.94 (0.76 to 1.17)	−0.90 (−3.79 to 1.99)		0.36 (0.28 to 0.47)	−13.45 (−16.42 to −10.48)		0.36 (0.25 to 0.50)	−14.90 (−19.37 to −10.43)	
Severe anaemia*	0	7 (0.8)	1 (0.1)	23 (2.9)	0.26 (0.09 to 0.72)	−0.77 (−1.50 to −0.04)		0.03 (0 to 0.31)	−1.69 (−2.46 to −0.92)		—	—	
Gestational weight gain, kg‡, mean (SD)	5.3 (2.6)	4.7 (2.6)	5.4 (2.7)	4.2 (2.5)	—	0.16 (−0.05 to 0.36)		—	0.88 (0.68 to 1.08)		—	1.08 (0.80 to 1.36)
Gestational weight gain, kg§, mean (SD)	8.8 (3.4)	7.7 (3.8)	9.1 (3.6)	7.3 (3.6)	—	0 (−0.28 to 0.29)		—	1.42 (1.15 to 1.70)		—	1.48 (1.10 to 1.86)
Morbidity during pregnancy and child birth (reported in first week after birth)												
Antepartum haemorrhage, n/total (%)*	2/1145 (0.2)	6/1004 (0.6)	2/959 (0.2)	4/856 (0.5)	1.09 (0.30 to 3.94)	0.03 (−0.39 to 0.45)		0.19 (0.04 to 0.77)	−0.35 (−0.81 to 0.12)		0.22 (0.03 to 1.85)	−0.29 (−0.92 to 0.34)
Preeclampsia or eclampsia, n/total (%)*	17/1008 (1.7)	33/876 (3.8)	24/894 (2.7)	35/788 (4.4)	0.72 (0.45 to 1.14)	−0.67 (−2.05 to 0.72)		0.55 (0.33 to 0.91)	−1.68 (−3.12 to −0.24)		0.37 (0.17 to 0.78)	−2.78 (−4.90 to −0.66)
Postpartum haemorrhage, n/total (%)*	16/1153 (1.4)	26/1012 (2.6)	29/969 (3.0)	23/859 (2.7)	0.68 (0.41 to 1.12)	−1.44 (−2.49 to −0.38)		0.83 (0.49 to 1.41)	−0.38 (−1.68 to 0.92)		0.47 (0.20 to 1.08)	−1.55 (−3.15 to 0.06)

Data are numbers (%) unless indicated otherwise.

ARR=absolute risk reduction; CI=confidence interval; IRR=incidence rate ratio; MD=mean difference; RTI=reproductive tract infection; SD=standard deviation.

* Incidence rate ratio, †mean difference, adjusted for place of birth, family possesses below poverty line card, woman’s height, woman’s body mass index for potential confounder and twins for clustering within the household. Not corrected for multiple outcomes or comparisons. ‡Weight gain between confirmation of pregnancy and 26-28 weeks of gestation (n=1086, 1004, 894, 840 for groups A-D, respectively); §weight gain between confirmation of pregnancy and 35-36 weeks of gestation (n=1117, 977, 934, 832 for groups A-D, respectively). ¶Absolute risk reduction, adjusted for place of birth, family possesses below poverty line card, woman’s height, woman’s body mass index for potential confounder and twins for clustering within the household.

Reproductive tract infection: any symptoms of vaginal discharge, itching, burning, swelling, ulcer in genital region, swelling and pain lower abdomen; hypertension: systolic blood pressure ≥140 mm Hg and diastolic blood pressure ≥90 mm Hg; no anaemia: haemoglobin ≥11g/dL; mild anaemia: haemoglobin 10-10.99 g/dL; moderate anaemia: haemoglobin 7-9.99 g/dL; severe anaemia: haemoglobin <7 g/dL; antepartum haemorrhage: reported excessive bleeding from vagina occurring any time during pregnancy, before delivery that wet her clothes; postpartum haemorrhage: reported excessive bleeding after delivery, with or without loss of consciousness; preeclampsia: measured diastolic blood pressure ≥90 mm Hg or systolic blood pressure ≥140 mm Hg and proteinuria (by dipstick1-4+); eclampsia: preclampsia with convulsions before, during, or after pregnancy.

Group A: preconception, pregnancy and early childhood intervention; group B: only preconception intervention; group C: only pregnancy and early childhood intervention; group D: control. Group A+B *v* group C+D: effect of preconception interventions: women who received interventions compared with those who did not receive preconception interventions; group A+C *v* group B+D: effect of pregnancy and early childhood interventions: women who received these interventions compared with those who did not receive these interventions; group A *v* group D: effect of preconception, pregnancy, and early childhood interventions: women who received interventions in these periods compared with those who received routine care.

### Pregnancy outcomes: primary

The proportion LBW was lower (incidence rate ratio 0.85, 98.3% confidence interval 0.75 to 0.97; absolute risk reduction −3.80%, 98.3% confidence interval −6.99% to −0.60%) and the proportion SGA was also lower (0.87, 0.78 to 0.98; −4.04%, −7.47% to −0.62%) in the preconception intervention groups (A+B) than in the groups that did not receive preconception interventions (C+D). Birth weight was higher (mean difference 40.84 g, 98.3% confidence interval 7.84 to 73.84 g) and birth length was also higher (0.17 cm, 0.01 to 0.32 cm) in the preconception intervention than in the no preconception intervention groups. The proportion of preterm births did not differ between the groups (incidence rate ratio 1.05, 98.3% confidence interval 0.87 to 1.27; absolute risk reduction 0.59%, 98.3% confidence interval −1.79% to 2.98%; table 5).

**Table 5:** Prespecified comparisons of primary and secondary outcomes at birth

Outcomes	Group A (n=1290)	Group B (n=1276)	Group C (n=1093)	Group D (n=1093)	Preconception intervention *v* no preconception intervention (A+B *v* C+D)			Pregnancy intervention *v* no pregnancy intervention (A+C *v* B+D)			Preconception and pregnancy intervention *v* no preconception and pregnancy intervention (A *v* D)	
					IRR (98.3% CI)	ARR¶ (%) or mean difference (98.3% CI)		IRR (98.3% CI)	ARR¶ (%) or mean difference (98.3% CI)		IRR (98.3% CI)	ARR¶ (%) or MD (98.3% CI)
**Primary**												
LBW*†	267/1141 (23.4)	239/1094 (21.9)	235/955 (24.6)	267/934 (28.6)	0.85 (0.75 to 0.97)	−3.80 (−6.99 to −0.60)		0.87 (0.76 to 1.01)	−1.71 (−4.96 to 1.54)		0.76 (0.62 to 0.91)	−5.59 (−10.32 to −0.85)
Preterm†	171/1283 (13.3)	158/1259 (12.6)	110/1080 (10.2)	160/1081 (14.8)	1.05 (0.87 to 1.27)	0.59 (−1.79 to 2.98)		0.85 (0.69 to 1.05)	−1.47 (−3.86 to 0.93)		0.91 (0.69 to 1.19)	−0.86 (−4.36 to 2.64)
SGA†	318/1138 (27.9)	325/1088 (29.9)	271/945 (28.7)	344/925 (37.2)	0.87 (0.78 to 0.98)	−4.04 (−7.47 to −0.62)		0.8 (0.71 to 0.90)	−6.60 (−10.15 to −3.05)		0.71 (0.61 to 0.83)	−11.84 (−16.94 to −6.75)
Birth weight (g), mean (SD)‡	n=1141, 2797 (438)	n=1094, 2816 (449)	n=955, 2785 (439)	n=934, 2744 (459)	—	40.84 (7.84 to 73.84)		—	35.97 (−0.17 to 72.12)		—	77.67 (26.38 to 128.96)
Birth length (cm), mean (SD)§‡	n=1161, 48.0 (2.1)	n=1102, 48.2 (2.1)	n=977, 48.0 (2.1)	n=940, 47.9 (2.2)	—	0.17 (0.01 to 0.32)		—	0.05 (−0.12 to 0.22)		—	0.21 (−0.03 to 0.45)
**Secondary**												
Spontaneous preterm†	99/1283 (7.7)	122/1259 (9.7)	62/1080, (5.7)	106/1081 (9.8)	1.14 (0.89 to 1.45)	0.94 (−1.00 to 2.88)		0.69 (0.53 to 0.90)	−2.93 (−4.88 to −0.98)		0.79 (0.55 to 1.12)	−2.00 (−4.77 to 0.76)
Stunting†	176/1161 (15.2)	176/1102 (16.0)	168/977 (17.2)	198/939 (21.1)	0.81 (0.69 to 0.96)	−3.18 (−5.91 to −0.45)		0.84 (0.70 to 1.00)	−2.10 (−4.88 to 0.67)		0.68 (0.53 to 0.87)	−5.32 (−9.42 to −1.22)
Head circumference (cm), mean (SD)‡	n=1161, 33.1 (1.3)	n=1102, 33.1 (1.3)	n=976, 33.1 (1.4)	n=940, 33.0 (1.4)	—	0.07 (−0.03 to 0.17)		—	0.12 (0.01 to 0.23)		—	0.18 (0.02 to 0.33)
Stillbirths†	22/1312 (1.7)	20/1296 (1.5)	15/1108, (1.4)	21/1114 (1.9)	0.97 (0.56 to 1.67)	0.01 (−0.89 to 0.87)		1.17 (0.62 to 2.18)	−0.26 (−1.16 to 0.64)		1.37 (0.59 to 3.19)	−0.26 (−1.55 to 1.04)

Data are n/total (%) unless indicated otherwise.

ARR=absolute risk reduction; CI=confidence interval; IRR=incidence rate ratio; MD=mean difference; SD=standard deviation.

* Weight on day 7 (up to +6 days). †Incidence rate ratio, ‡mean difference, adjusted for place of birth, family possesses below poverty line card, woman’s height, woman’s body mass index as potential confounders and twins for clustering within a household. 98.3% confidence interval for primary and secondary outcomes (to correct for multiple comparisons). §Length on days 1-7 (up to +6 days). ¶Absolute risk reduction, adjusted for place of birth, family possesses below poverty line card, woman’s height, woman’s body mass index for potential confounder and twins for clustering within the household.

Group A: preconception, pregnancy and early childhood intervention; group B: only preconception intervention; group C: only pregnancy and early childhood intervention; group D: control. Group A+B *v* group C+D: effect of preconception interventions: women who received interventions compared with those who did not receive preconception interventions; group A+C *v* group B+D: effect of pregnancy and early childhood interventions: women who received these interventions compared with those who did not receive these interventions; group A *v* group D: effect of preconception, pregnancy, and early childhood interventions: women who received interventions in these periods compared with those who received routine care.

The proportion SGA was lower in the pregnancy intervention groups than in the groups that did not receive pregnancy interventions (A+C *v* B+D; incidence rate ratio 0.80, 98.3% confidence interval 0.71 to 0.90; absolute risk reduction −6.60%, 98.3% confidence interval −10.15% to −3.05%). The proportion LBW (0.87, 0.76 to 1.01; −1.71, −4.96% to 1.54%) and proportion preterm birth (0.85, 0.69 to 1.05; −1.47%, −3.86% to 0.93%) were also lower in the pregnancy intervention than in the no pregnancy intervention groups, and the mean birth weight was also lower (mean difference 35.97 g, 98.3% confidence interval −0.17 to 72.12 g), but the upper limit of the confidence interval crossed null effect (table 5).

The proportion LBW was lower (incidence rate ratio 0.76, 98.3% confidence interval 0.62 to 0.91; absolute risk reduction −5.59%, 98.3% confidence interval −10.32% to −0.85%) and the proportion SGA was also lower (0.71, 0.61 to 0.83; −11.84%, −16.94% to −6.75%) in the group that received interventions during both preconception and pregnancy periods (A) than in the control group (D). Birth weight was 77.67 g higher (98.3% confidence interval 26.38 to 128.96 g); birth length (mean difference 0.21 cm, 98.3% confidence interval −0.03 to 0.45 cm) and proportion of preterm births (incidence rate ratio 0.91, 98.3% CI 0.69 to 1.19; absolute risk reduction −0.86%, 98.3% confidence interval −4.36% to 2.64%) did not differ in groups A and D (table 5).

### Pregnancy outcomes: secondary

The proportion stunted at birth was lower (incidence rate ratio 0.81, 98.3% confidence interval 0.69 to 0.96; absolute risk reduction −3.18%, 98.3% confidence interval −5.91% to −0.45%) in the preconception intervention groups (A+B) than in the groups that did not receive preconception interventions (C+D). Head circumference (mean difference 0.07, 98.3% confidence interval −0.03 to 0.17 cm) and still births (incidence rate ratio 0.97, 98.3% confidence interval 0.56 to 1.67; absolute risk reduction 0.01%, 98.3% confidence interval −0.89% to 0.87%) did not differ between the groups.

The proportion stunted at birth was lower (incidence rate ratio 0.84, 98.3% confidence interval 0.70 to 1.00; absolute risk reduction −2.10%, 98.3% confidence interval −4.88% to 0.67%) and head circumference was 0.12 cm higher (98.3% confidence interval 0.01 to 0.23 cm) in the pregnancy intervention groups (A+C) than in the groups that did not receive pregnancy interventions (B+D). Still births (incidence rate ratio 1.17, 98.3% confidence interval 0.62 to 2.18; absolute risk reduction −0.26%, 98.3% confidence interval −1.16% to 0.64%) did not differ between the groups (table 5).

The proportion stunted at birth was lower (incidence rate ratio 0.68, 98.3% confidence interval 0.53 to 0.87; absolute risk reduction −5.32%, 98.3% confidence interval −9.42 to −1.22) and head circumference was 0.18 cm higher (98.3% confidence interval 0.02 to 0.33 cm) in the preconception and pregnancy intervention group (A) than in the control group (D). Still births (1.37, 0.59 to 3.19; −0.26%, −1.55% to 1.04%) did not differ between the groups (table 5).

### Child growth from birth to 24 months of age

**Fig 2:**
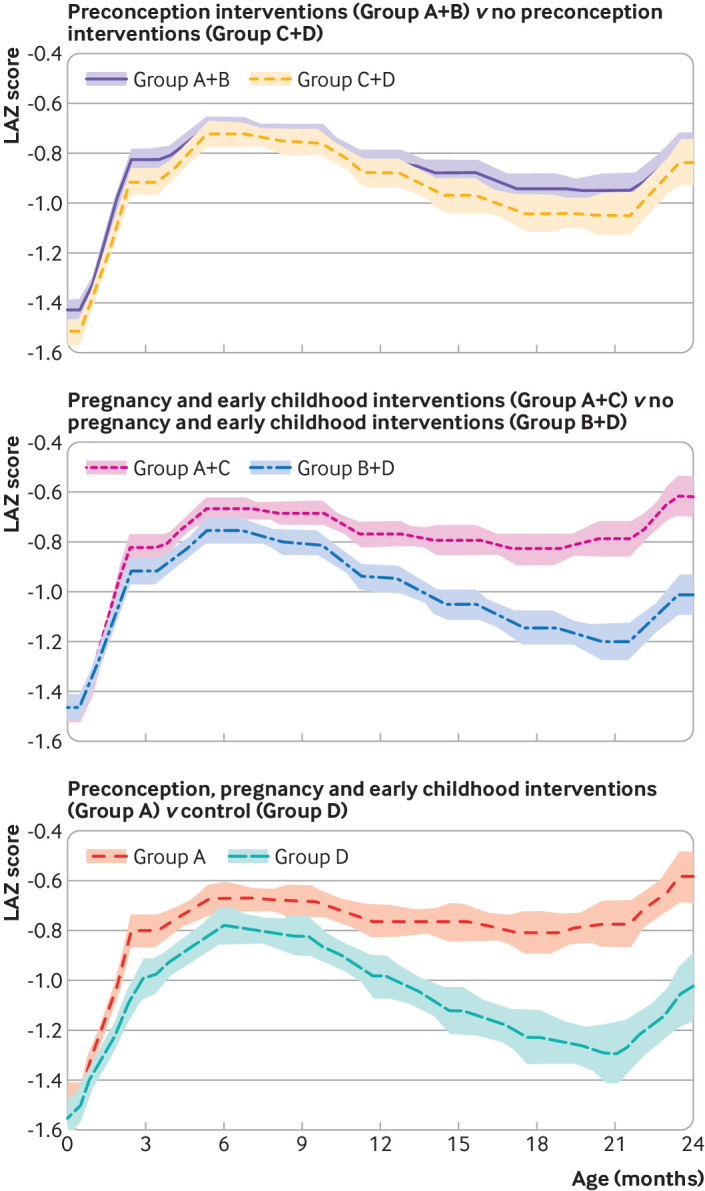
Mean length-for-age z (LAZ) scores from birth to 24 months; cross sectional data, shaded areas 95% confidence intervals. Group A: preconception, pregnancy and early childhood intervention; group B: only preconception intervention; group C: only pregnancy and early childhood intervention; group D: control. Upper panel, numbers at each age: group A+B—2263 at 0 month, 2219 at 3 months, 2259 at 6 months, 2058 at 9 months, 1874 at 12 months, 1563 at 15 months, 1298 at 18 months, 1061 at 21 months, 892 at 24 months; group C+D—1916 at 0 month, 1860 at 3 months, 1867 at 6 months, 1623 at 9 months, 1061 at 12 months, 1128 at 15 months, 887 at 18 months, 709 at 21 months, 564 at 24 months. Middle panel, numbers at each age: group A+C—2138 at 0 month, 2077 at 3 months, 2113 at 6 months, 1888 at 9 months, 1675 at 12 months, 1360 at 15 months, 1107 at 18 months, 903 at 21 months, 746 at 24 months; group B+D—2041 at 0 month, 2002 at 3 months, 2013 at 6 months, 1793 at 9 months, 1608 at 12 months, 1331 at 15 months, 1078 at 18 months, 867 at 21 months, 710 at 24 months. Lower panel, numbers at each age: group A—1161 at 0 month, 1137 at 3 months, 1160 at 6 months, 1053 at 9 months, 953 at 12 months, 787 at 15 months, 658 at 18 months, 540 at 21 months, 453 at 24 months; group D—939 at 0 month, 920 at 3 months, 914 at 6 months, 788 at 9 months, 687 at 12 months, 555 at 15 months, 438 at 18 months, 346 at 21 months, 271 at 24 months

The mean length-for-age z scores between birth and 24 months of age were higher from 9 months of age in the pregnancy and early childhood intervention groups (A+C *v* B+D), from 6 months of age in the groups that received preconception, pregnancy and early childhood interventions (A *v* D), but did not differ in the groups that received or did not receive preconception interventions (A+B *v* C+D; fig 2).

### Child outcomes at 24 months: primary

At 24 months of age, the proportion of children stunted (incidence rate ratio 0.96, 98.3% confidence interval 0.71 to 1.29; absolute risk reduction 0.69%, 98.3% confidence interval −3.39% to 4.78%) and the mean length-for-age z score (mean difference 0.08, 98.3% confidence interval −0.06 to 0.21) did not differ in the preconception intervention groups (A+B) and the groups that did not receive preconception interventions (C+D; table 6). The proportion stunted at 24 months of age was substantially lower (0.51, 0.38 to 0.70; −8.32%, −12.31% to −4.32%) and mean length-for-age z score was 0.40 standard deviation higher (98.3% confidence interval 0.27 to 0.54) in the pregnancy and early childhood intervention groups (A+C) than in the groups that did not receive these interventions (B+D; table 6). Similarly, the proportion stunted at 24 months of age was substantially lower (0.49, 0.32 to 0.75; −7.98%, −14.24% to −1.71%) and mean length-for-age z score was 0.46 standard deviation higher (0.27 to 0.66) in the group that received interventions during preconception, pregnancy, and early childhood (A) than in the control group (D; table 6).

**Table 6:** Primary and secondary anthropometry outcomes at 24 months of age

Outcomes	Group A (n=453)		Group B (n=439)	Group C (n=293)	Group D (n=271)	Preconception intervention *v* no preconception intervention (group A+B *v* C+D)				Pregnancy intervention *v* no pregnancy intervention (group A+C *v* B+D)			Preconception and pregnancy intervention *v* no preconception and pregnancy intervention (A *v* D)	
						IRR (98.3% CI)		ARR‡ (%) or MD (98.3% CI)		IRR (98.3% CI)	ARR‡ (%) or MD (98.3% CI)		IRR (98.3% CI)	ARR‡ (%) or MD (98.3% CI)
**Primary outcomes**														
Length-for-age z score, mean (SD)*	−0.6 (1.2)	−1.0 (1.1)		−0.7 (1.1)	−1.0 (1.2)	—	0.08 (−0.06 to 0.21)			—	0.40 (0.27 to 0.54)		—	0.46 (0.27 to 0.66)
Stunted†	47 (10.4)	85 (19.4)		32 (10.9)	51 (18.8)	0.96 (0.71 to 1.29)	0.69 (−3.39 to 4.78)			0.51 (0.38 to 0.70)	−8.32 (−12.31 to −4.32)		0.49 (0.32 to 0.75)	−7.98 (−14.24 to −1.71)
**Secondary outcomes**														
Weight-for-length z score, mean (SD)*	−0.7 (1.1)	−1.1 (1.0)		−0.8 (1.1)	−1.1 (1.0)	—	0.05 (−0.09 to 0.18)			—	0.33 (0.20 to 0.47)		—	0.40 (0.20 to 0.60)
Wasted†	54 (11.9)	71 (16.2)		32 (10.9)	43 (15.9)	1.04 (0.75 to 1.43)	1.01 (−3.15 to 5.16)			0.68 (0.49 to 0.96)	−5.12 (−9.18 to −1.06)		0.71 (0.44 to 1.13)	−3.71 (−9.57 to 2.15)
Weight-for-age z score, mean (SD)*	−0.8 (1.2)	−1.3 (1.1)		−0.9 (1.1)	−1.3 (1.2)	—	0.08 (−0.06 to 0.22)			—	0.45 (0.31 to 0.59)		—	0.54 (0.33 to 0.75)
Underweight†	70 (15.5)	104 (23.7)		42 (14.3)	67 (24.7)	0.97 (0.76 to 1.25)	0.78 (−3.80 to 5.36)			0.57 (0.44 to 0.75)	−9.17 (−13.66 to −4.68)		0.56 (0.39 to 0.80)	−8.76 (−15.67 to −1.85)
Mid upper arm circumference (cm), mean (SD)*	14.8 (1.3)	14.4 (1.1)		14.7 (1.2)	14.4 (1.1)	—	0.08 (−0.07 to 0.23)			—	0.39 (0.24 to 0.54)		—	0.49 (0.28 to 0.71)
Head circumference (cm), mean (SD)*	46.1 (1.4)	45.8 (1.4)		45.9 (1.5)	45.7 (1.4)	—	0.14 (−0.05 to 0.32)			—	0.34 (0.15 to 0.53)		—	0.46 (0.19 to 0.74)

Data are numbers (%) unless indicated otherwise.

ARR=absolute risk reduction; CI=confidence interval; IRR=incidence rate ratio; MD=mean difference; SD=standard deviation.

* Mean difference, †incidence rate ratio, adjusted for place of birth, family possesses below poverty line card, woman’s height, woman’s body mass index, as potential confounders and twins for clustering within a household; 98.3% CI for primary and secondary outcomes (to correct for multiple comparisons). **‡**Absolute risk reduction, adjusted for place of birth, family possesses below poverty line card, woman’s height, woman’s body mass index for potential confounder and twins for clustering within the household.

Stunting: length-for-age z score <–2 standard deviations of World Health Organization child growth standards; wasting: weight-for-height z score <−2 standard deviations of WHO child growth standards; underweight: weight-for-age z score <−2 standard deviations of WHO child growth standards. Group A: preconception, pregnancy and early childhood intervention; group B: only preconception intervention; group C: only pregnancy and early childhood intervention; group D: control. Group A+B *v* group C+D: effect of preconception interventions: women who received interventions compared with those who did not receive preconception interventions; group A+C *v* group B+D: effect of pregnancy and early childhood interventions: women who received these interventions compared with those who did not receive these interventions; group A *v* group D: effect of preconception, pregnancy, and early childhood interventions: women who received interventions in these periods compared with those who received routine care.

### Child outcomes at 24 months: secondary

At 24 months of age, weight-for-length z score (mean difference 0.05, 98.3% confidence interval −0.09 to 0.18) and weight-for-age z score (0.08, −0.06 to 0.22) did not differ in the preconception intervention groups (A+B) and the groups that did not receive preconception interventions (C+D). Similarly, wasting (incidence rate ratio 1.04, 98.3% confidence interval 0.75 to 1.43; absolute risk reduction 1.01%, 98.3% confidence interval −3.15% to 5.16%), underweight (0.97, 0.76 to 1.25; 0.78%, −3.80% to 5.36%), and head circumference (mean difference 0.14, 98.3% confidence interval −0.05 to 0.32) did not differ between these groups (table 6).

The mean weight-for-length z score was 0.33 standard deviation higher (98.3% confidence interval 0.20 to 0.47), the mean weight-for-age z score was 0.45 standard deviation higher (0.31 to 0.59), and the head circumference was 0.34 cm higher (0.15 to 0.53) in the pregnancy and early childhood intervention groups (A+C) than in the groups that did not receive these interventions (B+D). Similarly, the proportion who were wasted was lower (incidence rate ratio 0.68, 98.3% confidence interval 0.49 to 0.96; absolute risk reduction −5.12%, 98.3% confidence interval −9.18% to −1.06%) and the proportion underweight was lower (0.57, 0.44 to 0.75; −9.17%, −13.66% to −4.68%) in groups A+C than in groups B+D (table 6).

The mean weight-for-length z score was 0.40 standard deviation higher (98.3% confidence interval 0.20 to 0.60), the mean weight-for-age z score was 0.54 standard deviation higher (0.33 to 0.75), and the head circumference was 0.46 cm higher (0.19 to 0.74) in the group that received interventions during preconception, pregnancy and early childhood (A) than in the control group (D). Similarly, the proportion who were wasted was lower (incidence rate ratio 0.71, 98.3% confidence interval 0.44 to 1.13; absolute risk reduction −3.71%, 98.3% confidence interval −9.57% to 2.15%) and the proportion underweight was lower (0.56, 0.39 to 0.80; −8.76%, −15.67% to −1.85%) in group A than in group D (table 6).

We assessed the interaction between preconception interventions and pregnancy and early childhood interventions for all primary outcomes and present comparisons of individual groups (table 7). We did not find any evidence of interactions except for the effect on preterm birth. This analysis is generally consistent with the results of the prespecified comparisons. Preconception interventions had important effects on birth size but not on linear growth at 24 months compared with the control group. Pregnancy and early childhood interventions had important effects on birth size and on linear growth at 24 months compared with the control group. The effect sizes of preconception and pregnancy and early childhood interventions together were larger for birth size and linear growth at 24 months than those of the control group compared with the effect sizes of interventions provided only in a single period.

**Table 7:** Interaction between groups that received preconception or pregnancy interventions compared with those that did not receive these interventions

Primary outcome	No preconception intervention			Preconception intervention		Measure of interaction (95% CI)
	% or mean (SD)	IRR or MD (95% CI)		% or mean (SD)	IRR or MD (95% CI)	
**Low birth weight**						
No pregnancy intervention	28.6	1.00		21.9	0.78 (0.67 to 0.91); P=0.001	1.20 (0.97 to 1.48); P=0.10
Pregnancy intervention	24.6	0.82 (0.69 to 0.96); P=0.01		23.4	0.76 (0.65 to 0.88); P<0.001	
**Preterm birth**						
No pregnancy intervention	14.8	1.00		12.6	0.88 (0.71 to 1.09); P=0.24	1.52 (1.11 to 2.08); P=0.01
Pregnancy intervention	10.2	0.69 (0.54 to 0.89); P=0.005		13.3	0.91 (0.73 to 1.14); P=0.41	
**Small for gestational age**						
No pregnancy intervention	37.2	1.00		29.9	0.81 (0.72 to 0.91); P=0.001	1.17 (0.98 to 1.40); P=0.09
Pregnancy intervention	28.7	0.76 (0.66 to 0.87); P<0.001		27.9	0.71 (0.62 to 0.81); P<0.001	
**Birth weight (g)**						
No pregnancy intervention	2744 (459)	0.00		2816 (449)	65 (26 to 103); P=0.001	−47 (−101 to −7); P=0.09
Pregnancy intervention	2785 (439)	60 (17 to 103); P=0.007		2797 (438)	77 (36 to 119); P<0.001	
**Birth length (cm)**						
No pregnancy intervention	47.9 (2.2)	0.00		48.19 (2.07)	0.29 (0.11 to 0.47); P=0.002	−0.23 (−0.48 to 0.02); P=0.08
Pregnancy intervention	48.0 (2.1)	0.21 (−0.01 to 0.41); P=0.05		47.98 (2.06)	0.23 (0.02 to 0.44); P=0.03	
**Length-for-age z score at 24 months**						
No pregnancy intervention	−1.0 (1.2)	0.00		−1.00 (1.13)	0.01 (−0.15 to 0.17); P=0.90	0.12 (−0.10 to 0.34); P=0.28
Pregnancy intervention	−0.7 (1.1)	0.31 (0.14 to 0.48); P<0.001		−0.58 (1.17)	0.46 (0.30 to 0.63); P<0.001	
**Stunting at 24 months**						
No pregnancy intervention	18.8	1.00		19.4	0.98 (0.73 to 1.32); P=0.91	0.92 (0.56 to 1.54); P=0.76
Pregnancy intervention	10.9	0.56 (0.37 to 0.84); P=0.005		10.4	0.49 (0.34 to 0.70); P<0.001	

CI=confidence interval; IRR=incidence rate ratio; MD=mean difference; SD=standard deviation.

### Post hoc sensitivity analysis adjusting for multiple primary outcomes and comparisons

After post hoc adjustment for seven primary outcomes in addition to the three comparisons using the Holm-Bonferroni method, statistically significant differences remained for all measures except mean birth length in the preconception intervention groups (A+B) compared with the groups that did not receive these interventions (C+D; Holm-Bonferroni adjusted P=0.09; table S4, supplementary appendix).

### Subgroup analyses for primary outcomes

The subgroup analyses for primary outcomes for all prespecified comparisons did not show any significant effect modification (figs S1-S7, supplementary appendix). The intervention impact at birth and at 24 months was similar for short (<150 cm) and tall (≥150 cm) women.

### Other secondary outcomes

Findings of other secondary outcomes related to the mothers and their children are shown in the supplementary appendix (fig S7, tables S10-S13). One noteworthy finding was that the proportion of infants exclusively breastfed at 5 months of age was higher (incidence rate ratio 2.78, 98.3% confidence interval 2.54 to 3.05; absolute risk reduction 47.68%, 98.3% confidence interval 44.20% to 51.15%) in the pregnancy and early childhood intervention groups (A+C *v* B+D), and in the group that received interventions in both periods (group A *v* D; 2.57, 2.25 to 2.92; 45.15%, 40.11% to 50.19%).

Tables S14 and S15 in the supplementary appendix show the numbers of deaths in women and children . No adverse events related to the intervention were reported.

## Discussion

### Principal findings

In this trial, a package of health, nutrition, psychosocial care, and WaSH interventions delivered during preconception and pregnancy periods reduced the risk of LBW by 24%, more than half of which was attributed to preconception interventions. The intervention package delivered during preconception, pregnancy, and early childhood reduced the risk of stunting at two years of age by 51%; almost all the effect can be attributed to pregnancy and early childhood interventions. These intervention effects were not modified by maternal height. In addition to the effect on primary and secondary infant outcomes, the interventions improved several maternal outcomes—higher haemoglobin concentration and gestational weight gain, and reduced risk of reproductive tract infection, anaemia, and pregnancy induced hypertension.

### Comparison with other studies

In this large trial, interventions in several domains were delivered concurrently and LBW and stunting related outcomes were assessed. We will briefly summarise the results of previous trials. Nutrition interventions during pregnancy such as supplementing with multiple micronutrients and balanced protein energy have been shown to reduce LBW by 12% in low and middle income countries, but not SGA.^28^ ^29^ ^30^ However, the role of infection related interventions is unclear.^31^ Evidence is lacking about the impact of preconception health or nutrition interventions on LBW, SGA, and preterm births.^32^ ^33^ ^34^ Complementary food supplementation was shown to have a small effect on length-for-age z score (standardised mean difference 0.08, 95% confidence interval 0.04 to 0.13) in children aged 6-23 months in food insecure settings.^35^ WaSH interventions and complementary feeding did not improve child growth compared with feeding interventions alone.^36^ Integrated care giving and nutrition interventions improved weight-for-length z score but not weight-for-age and length-for-age z scores.^37^

Fortified lipid based nutrient supplements containing 220-285 kcal during the complementary feeding age have been found to reduce the risk of stunting (relative risk 0.80, 95% credible interval 0.66 to 0.97) compared with the standard of care in a network meta-analysis.^38^ Education interventions focusing on the appropriate introduction of complementary feeding have been found to increase infant weight and length at six months and older, and reduce the risk of undernutrition in term infants.^39^ ^40^ ^41^ A cluster randomised study in Zambia found improved length-for-age z scores in children who were stunted with home based growth monitoring (0.50, 95% confidence interval 0.165 to 0.85) and community based growth monitoring and nutritional supplementation (0.58, 0.13 to 1.03) during the complementary feeding period compared with the control group.^42^ A recent network meta-analysis suggested that multiple micronutrient supplementation in apparently healthy infants improves linear growth (length-for-age z score: mean difference 0.20, 95% credible interval 0.03 to 0.35) in the first six months of life.^38^

Several mechanisms in the WHO framework on childhood stunting explain our findings related to LBW and stunting.^43^ Preconception interventions improved anaemia, nutritional status, and reduced the risk of reproductive tract infection, which might increase fertility. A higher proportion of live births was found in the preconception intervention group than in the no preconception intervention group. The possible reasons could be, firstly, preconception interventions improved anaemia and nutritional status and reduced the risk of reproductive tract infections, which might have improved fertility among women in this group. Secondly, women in the preconception intervention group received counselling on positive thinking and problem solving skills, which might have led to a state of improved mental wellbeing, conducive to plan for a pregnancy. Pregnancy interventions increased gestational weight gain, reduced the risk of anaemia, micronutrient deficiency, reproductive tract infection, and pregnancy induced hypertension; these are major contributors to LBW. Early childhood interventions improved breastfeeding and complementary feeding, childcare practices, and maternal nutritional status, which are key contributors of stunting.

While the preconception interventions were at least as important as pregnancy interventions for birth outcomes, the effect was diluted two years after birth when preconception interventions only had a marginal additional benefit to pregnancy and early childhood interventions on the outcomes at 24 months. One possible explanation could be that intensive breastfeeding counselling, provision of high quality complementary food, and early child play stimulation after birth could play critical parts in improving child growth. Delivering interventions using several home visits might also have contributed to high compliance rates. We hypothesise that a complex, multifactorial problem like stunting could only be addressed by a complex intervention addressing key health, nutrition, psychosocial care, and environment issues at the same time. This theory is shown by the larger impact of our intervention compared with that seen in studies examining simple interventions. Our results are particularly generalisable to low and middle income populations in South Asia.

### Strengths and limitations of this study

The strengths include a rigorous study design, large sample size, high compliance for most interventions, well standardised outcome measurements, early pregnancy ultrasound based gestation assessment, and generalisability to low and middle income urban populations.

Some limitations need consideration. We could not use community mobilisation to promote interventions because of the individually randomised design. Intensity of delivery of a few interventions such as psychosocial support, management of chronic disease in women, and common illnesses in children was lower than desired. Anthropometry outcomes were assessed at day 7 (+6 days) after birth to ensure similar outcome assessment across study groups given the greater access of study teams to intervention group mothers immediately after birth. Lockdowns due to the covid-19 pandemic affected intervention delivery and delayed outcome assessment in a few instances.

Additionally, the study could not be blinded because of the nature of interventions; however, study outcomes were assessed by an independent team not involved in delivering interventions or informed of the group allocation before measurements. We do not believe that the higher proportion of live births in the preconception intervention group compared with the no preconception intervention group caused any appreciable bias. It is possible that this might have attenuated the effect of preconception interventions because women in the preconception interventions group became pregnant earlier (median time between enrolment and pregnancy confirmation 126 days, interquartile range 33-275 days) than those in the no preconception intervention group (162 days, 50-311 days). The desired sample size at 24 months could not be achieved because the DSMB recommended stopping the study based on strong evidence for the beneficial effects for 24 month outcomes.

### Policy implications

The feasibility of implementing this impactful but complex intervention in routine programme settings needs to be carefully considered. While most interventions provided during pregnancy and early childhood are part of several national programmes, they need to be high quality. Research on how to implement these interventions within routine systems to improve adverse growth outcomes like stunting seems necessary and should be a priority.

### Conclusions

Our findings provide evidence that preconception interventions have major benefits for reducing the burden of infants with LBW and SGA. Strengthening the existing antenatal and early childhood programmes would reduce the risk of preterm births, SGA, and stunting at 24 months. Implementation research studies are important in other low and middle income countries to assess the feasibility of delivering intervention packages effectively, identify context specific barriers, monitor quality and coverage of programmes, and improve community awareness and rollout. Integrating preconception interventions into current health systems coupled with early identification and management of pregnant women and infants who are at high risk should be a priority. These findings provide an opportunity for policymakers and managers to review and improve current programmes to improve women’s health and reduce adverse pregnancy outcomes and the burden of undernutrition in children younger than 24 months of age.

**What is already known on this topic**

Evidence suggests no clear impact of preconception interventions on low birth weight, preterm birth, and small for gestational age or stunting at 24 months of age
Most studies have tested the effect of individual interventions after identifying pregnancy up to two years after birth
These interventions covered health, nutrition, water, sanitation and hygiene, and psychosocial health, with modest effects on birth weight and linear growth at 24 months of age

**What this study adds**

This study shows that a package of health, nutrition, psychosocial care, and water, sanitation and hygiene interventions delivered during preconception, pregnancy, and early childhood reduces the risk of low birth weight and stunting at 24 months of age
Preconception interventions had an important impact on birth outcomes but not on outcomes at 24 months of age
Preconception and pregnancy interventions improved several maternal outcomes—higher haemoglobin concentration and gestational weight gain, and reduced risk of reproductive tract infections, moderate to severe anaemia, and pregnancy induced hypertension

## Data Availability

The organisation conducting the trial (Society for Applied Studies, India) is a collaborator in the Healthy Birth, Growth, and Development Knowledge Integration (HBGDKi) of the Bill and Melinda Gates Foundation and the data generated from the study will be shared as part of the HBGDKi repository (https://github.com/HBGDki). Individual requests will also be considered on a case-by-case basis. The request for data should be accompanied by a detailed proposal describing the scientific questions to be addressed. Proposals should be submitted to NB (nita.bhandari@sas.org.in).

## Web extra

Extra material supplied by authors

Web appendix: supplementary appendix

## Notes

Contributors: All authors were involved in proposal development, study design, and periodic reviews of study activities. They also led specific activities: ST was responsible for study implementation and design and development of the data management system, RC for study implementation, quality control and monitoring, and ND led the team providing medical management to enrolled participants. SM, RPU, and SS led the development of WaSH, psychosocial care, and childhood interventions, respectively. RD, PM, and HC were responsible for management of study participants in the collaborating hospital. NB was responsible for overall study implementation and provided guidance to the study teams on an ongoing basis. RB and MKB provided technical guidance. KB, RC, ST, and RB conducted the analyses, vouch for the data, and the analyses presented. The data were gathered by different study teams. The authors in the study group were responsible for day-to-day oversight of different activities. NB and RB contributed equally to the manuscript. ST, RC, NB, and RB wrote the first draft of the manuscript. All authors reviewed and finalised the manuscript. ST, RC, and NB are responsible for the overall content as guarantors and accept full responsibility for the conduct of the study, had access to the data, and controlled the decision to publish. The corresponding author attests that all listed authors meet authorship criteria and that no others meeting the criteria have been omitted.

The lead author (the manuscript’s guarantor) affirms that the manuscript is an honest, accurate, and transparent account of the study being reported; that no important aspects of the study have been omitted; and that any discrepancies from the study as originally planned (and, if relevant, registered) have been explained.

Dissemination to participants and related patient and public communities: The study findings will be disseminated through research publications in international and national peer reviewed journals, presentations in national and international conferences, and social media platforms. The authors will also organise meetings to share the results with the study participants and community leaders, and will engage with policymakers, national and state authorities for scaling up the interventions.

Provenance and peer review: Not commissioned; externally peer reviewed.
