# Supplementary material for: Impact of a package of health, nutrition, psychosocial support, and WaSH interventions delivered during preconception, pregnancy, and early childhood periods on birth outcomes and on linear growth at 24 months of age: factorial, individually randomised controlled trial
Source: BMJ. 2022 Oct 26;379:e072046. doi: 10.1136/bmj-2022-072046 (PMC9597398; doi:10.1136/bmj-2022-072046)
Supplement: Supplementary file 1 — Web appendix: supplementary appendix [file tans072046.ww.pdf]

Impact of a package of health, nutrition, psychosocial support, and water, sanitation and hygiene interventions delivered during preconception, pregnancy and early childhood periods on birth outcomes and on linear growth at 24 months of age: a factorial, individually randomized controlled trial

SUPPLEMENTARY APPENDIX

TABLE OF CONTENTS

**Methods**	
Table S1. Control Group: National Programs for women of reproductive age, pregnant women and under-twos……………………………………………………	3
Table S2. Study interventions………………………………………………………..	5
Table S3. Secondary outcomes in women and children and timing of measurement ………………………………………………………………………	11
**Results**	
Figure S1. Subgroup analysis for low birth weight comparing preconception interventions and No preconception interventions (Group A+B vs Group C+D)…	13
Figure S2. Subgroup analysis for low birth weight comparing pregnancy and early childhood interventions and No pregnancy and early childhood interventions (Group A+C vs Group B+D)…...……………………………………………………….	14
Figure S3. Subgroup analysis for low birth weight comparing preconception and pregnancy and early life interventions and No preconception and pregnancy and early childhood interventions (Group A vs Group D)…………………………………	15
Figure S4. Subgroup analysis for stunting at 24 months comparing preconception interventions and No preconception interventions (Group A+B vs Group C+D)….	16
Figure S5. Subgroup analysis for stunting at 24 months comparing pregnancy and early childhood interventions and No pregnancy and early childhood interventions (Group A+C vs Group B+D)……………………………………………………………	17
Figure S6. Subgroup analysis for stunting at 24 months comparing preconception and pregnancy and early childhood interventions and No preconception and pregnancy and early childhood interventions (Group A vs Group D)……………….	18
Figure S7. Weight-for-age z-scores from birth to 24 months; cross-sectional data..	19
Table S4. Sensitivity analysis for primary outcomes at birth and 24 months………	20
Table S5. Compliance to interventions during the preconception period………….	21
Table S6. Compliance to interventions during pregnancy…………………………..	22
Table S7. Compliance to interventions in mothers during the postnatal period….	23
Table S8. 24-hour dietary recalls in a randomly selected subset of women during preconception, pregnancy and postpartum periods …………………………………	24
Table S9. Compliance to interventions in children from birth to 24 months………	25
Table S10. Secondary outcomes: Anthropometry at 6 and 12 months of age…….	26
Table S11. Secondary outcomes: Breastfeeding at infant ages 1, 5 and 12 months	28
Table S12. Secondary outcomes: Dietary intake data at infant ages 9, 12, 18 and 24 months…………………………………………………………………………….........	29
Table S13. Secondary outcomes: Morbidity at infant ages 1, 3, 6, 12, 18 and 24 months………………………………………………………………………………	30
Table S14. Number of women who died in the preconception period……………..	33
Table S15. Number of mothers and children who died during pregnancy or in the early childhood period………………………………………………………………..	34

Table S1. Control Group: National Programs for women of reproductive age, pregnant women and under-twos

The control group had access to services provided by Accredited Social Health Activists (https://nhm.gov.in/index1.php?lang=1&level=1&sublinkid=150&lid=226), Integrated Child Development Services (http://www.wcddel.in/icds.html), Municipal Corporation of Delhi (MCD) Health Centers, hospitals and private health care practitioners in and around the study areas:

Interventions	National Programs	Access from
**Preconception period**		
Family planning services including promotion of spacing methods	Reproductive, Maternal, Newborn, Child, and Adolescent Health (https://nhm.gov.in/images/pdf/RMNCH+A/RMNCH+A_Strategy.pdf), Mission *Parivar Vikas* (https://main.mohfw.gov.in/sites/default/files/06Chapter.pdf)	Community health workers called Accredited Social Health Activists (ASHAs), Maternity & Child Welfare Centres, Family Planning Units in government and private health facilities
Weekly iron-folic acid supplementation	Anemia *Mukt Bharat* (https://anemiamuktbharat.info/wp-content/uploads/2019/09/Anemia-Mukt-Bharat-Brochure_English.pdf)	*Anganwadi* centres, government dispensaries
Pregnancy period		
Early registration of pregnancy, four antenatal care check-ups, institutional delivery	My Safe Motherhood (https://nhm.gov.in/images/pdf/programmes/maternal-health/guidelines/my_safe_motherhood_booklet_english.pdf)	Public and private helath facilities
Daily iron-folic acid supplementation for 180 days	Anemia *Mukt Bharat* (https://anemiamuktbharat.info/wp-content/uploads/2019/09/Anemia-Mukt-Bharat-Brochure_English.pdf)	*Anganwadi* centres, government dispensaries, government and private health facilities
Daily calcium and vitamin D supplementation for 180 days	National Guidelines for Calcium Supplementation During Pregnancy and Lactation (http://www.nrhmhp.gov.in/sites/default/files/files/NG_calcium.pdf)	*Anganwadi* centres, government dispensaries, government and private health facilities
Single dose of tablet Albendazole (400 mg) after 1st trimester		*Anganwadi* centres, government dispensaries, government and private health facilities
Supplementary food for mothers, either cooked food or take-home ration (600 calories, 18-20 g of protein)	Integrated Child Development Services (http://www.wcddel.in/icds.html)	*Anganwadi* centres
Postnatal period		
Supplementary food for mothers, either cooked food or take-home ration (600 calories, 18-20 g of protein)	Integrated Child Development Services (http://www.wcddel.in/icds.html)	*Anganwadi* centres
Daily iron-folic acid supplementation for 180 days	Anemia *Mukt Bharat* (https://anemiamuktbharat.info/wp-content/uploads/2019/09/Anemia-Mukt-Bharat-Brochure_English.pdf	*Anganwadi* centres, government dispensaries, government and private health facilities
Daily calcium and vitamin D supplementation for 180 days	National Guidelines for Calcium Supplementation During Pregnancy and Lactation (http://www.nrhmhp.gov.in/sites/default/files/files/NG_calcium.pdf)	*Anganwadi* centres, government dispensaries, government and private health facilities

Interventions	National Programs	Access from
Postnatal health checkup	My Safe Motherhood (https://nhm.gov.in/images/pdf/programmes/maternal-health/guidelines/my_safe_motherhood_booklet_english.pdf)	Public and private health facilities where the woman has had her delivery
Postnatal period for children		
Promotion of optimal breastfeeding practices (early initiation of breastfeeding within one-hour, exclusive breastfeeding for the first six months, and continued breastfeeding for at least two years)	Mother's Absolute Affection –MAA (https://nhm.gov.in/MAA/Operational_Guidelines.pdf)	Home visits by ASHAs and *Anganwadi* workers
Home visits for newborn care (days 3, 7, 14, 21, 28 and 42; additionally, on day 1 for home births)	Home Based Newborn Care (http://www.nihfw.org/Doc/NCHRC-Publications/Operational%20Guidelines%20on%20Home%20Based%20Newborn%20Care%20%28HBNC%29.pdf)	ASHAs
Supplementary food (6 to 72 months of age): 500 calories, 12-15 g of protein. For children with severe acute malnutrition, supplementary food increased to 800 calories, 20-25 g of protein	Integrated Child Development Services (http://www.wcddel.in/icds.html)	*Anganwadi* centres
Immunization	Universal Immunization program (https://main.mohfw.gov.in/sites/default/files/5628564789562315.pdf) based on national immunization schedule (https://nhm.gov.in/New_Updates_2018/NHM_Components/Immunization/report/National_%20Immunization_Schedule.pdf)	Government dispensaries, public and private health facilities
IFA supplementation from 6 months to 24 months	Anemia *Mukt Bharat* (https://anemiamuktbharat.info/wp-content/uploads/2019/09/Anemia-Mukt-Bharat-Brochure_English.pdf)	*Anganwadi* centres, government dispensaries, private practitioners, public and private health facilities
Care for pneumonia and diarrhea among children Integrated Action Plan for Pneumonia and Diarrhea	SAANS initiative (https://nhm.gov.in/index1.php?lang=1&level=4&sublinkid=1336&lid=716)	Government dispensaries, public and private health facilities

Table S2. Study Interventions

PRECONCEPTION PERIOD

<table>
<tr><th>Intervention</th><th>Control‡</th></tr>
<tr><td>HEALTH
Screen and treat medical conditions known to affect fetal and infant growth i.e. reproductive tract infections, syndromic approach (RTI), symptoms of tuberculosis (TB), hypertension, diabetes (HbA1c ≥6.5%) and pre-diabetes (HbA1c 5.7%-6.4%), hypo-thyroidism (thyroid-stimulating hormone (TSH) >5.5 mIU/ml or TSH 4.01 to 5.5 mIU/l and anti-TPO Ab positive) and hyper-thyroidism (<0.4 mIU/ml)
Provision of contraception to women living with husband for <1 year, having a child aged <1 year, severe malnutrition, moderate to severe anemia, hypothyroidism, RTI/STI and diabetes, hypertension
Bi-annual deworming (Albendazole 400 mg)</td><td rowspan="4">Weekly IFA supplementation as part of the National Iron plus Initiative Program</td></tr>
<tr><td>NUTRITION
Weekly iron-folic acid (IFA) supplementation (300 mg ferrous fumerate, 1.5 mg folic acid, 15 mcg cyanocobalamin)
Multiple micronutrients thrice weekly*
Screening for malnutrition and anemia
Management of undernutrition
- BMI <16 kg/m^2: Refer to hospital, locally-prepared snacks and egg or milk† (1000 kcal/day and 20-22 g protein/day)
- BMI 16 to 18.49 kg/m^2: Snacks and egg or milk† (500 kcal/day and 12-14 g protein/day)
- BMI <18.5 to 20.99 kg/m^2: Egg or milk† (70 kcal, 6 g protein) 6 days a week
Monthly weight assessment for those with BMI <18.5 kg/m^2: nutrition counselling and management of infections for those with inadequate weight gain (IWG, weight gain <500 g per month)
Anemia
- Severe anemia (hemoglobin <8 g/dL): Refer to hospital for treatment
- Mild to moderate anemia (hemoglobin 8 to 11.99 g/dL): treatment with IFA (300 mg ferrous fumerate, 1.5 mg folic acid, 15 mcg cyanocobalamin)</td></tr>
<tr><td>WaSH
Promotion of personal, menstrual and hand hygiene</td></tr>
<tr><td>PSYCHOSOCIAL SUPPORT
Screen for depressive symptoms (using PHQ-9).
Counselling by study psychologist if PHQ score ≥10. Referral to psychiatrist if PHQ-9 score ≥15 and/or presence of suicidal ideation.
Promotion of positive thinking and problem-solving skills
Screen for substance abuse and exposure to second-hand smoke and alcohol use in husbands and counsel</td></tr>
</table>

*Composition given at the end

†Observed intake

Monitor above interventions every three months until corrected or pregnancy reported., Repeat investigations done at baseline and for those non-pregnant 12 months post-enrollment.

Electronic monitoring system for tracking women with problems to support them for achieving intervention compliance across all domains.

PREGNANCY

Intervention Group	Control Group[‡]
**HEALTH**	
At least eight antenatal care (ANC) contacts according to WHO ANC guidelines, registration for institutional delivery	
In addition to hospital-based ANC clinics, increase coverage through free-of-cost, high-quality, study outpatient clinic services within community including laboratory services.	
**Screening and treatment for medical conditions**:	
HIV, VDRL, syndromic RTI, syndromic TB, Hepatitis B (HbsAg), hypo- and hyper-thyroidism (TSH) assessment at first contact	
Urine routine and microscopic examination and asymptomatic bacteriuria by urine culture four times	
Gestational diabetes by oral glucose tolerance test thrice	
Pregnancy induced hypertension (blood pressure) at every visit	
Anemia (hemoglobin) four times	
Tetanus toxoid immunization	
Calcium (1000 mg) and vitamin D (400 IU) supplementation daily starting from second trimester throughout pregnancy	
Anti-helminthics (Albendazole 400 mg) at 20 weeks	
**NUTRITION**	
Counselling	
IFA (100 mg iron, 500 µg folic acid) supplementation daily throughout pregnancy from second trimester	Routine antenatal care
Multiple micronutrients* daily throughout pregnancy	
Food Supplements	
BMI <25 kg/m^2	
- Second trimester: Food supplements (210 kcal, 2 g protein) in the form of a choice of snacks prepared locally and milk (70 Kcal, 6 g protein)[†]	
- Third trimester: Food supplements (400 kcal, 21 g protein) in the form of a choice of snacks prepared locally and milk (70 Kcal, 6 g protein)[†]	
Extra snacks (500 kcal, 20 g protein) throughout pregnancy, to women with BMI <18.5 kg/m^2	
BMI 25 to <30 kg/m^2	
- Milk (70 kcal, 6 g protein) 6 days a week[†]	
No food supplements to overweight and obese women (BMI ≥25 kg/m^2)	
Anemia	
- Severe anemia (hemoglobin <7 g/dL): Refer to hospital for treatment	
- Mild to moderate anemia (hemoglobin 7 to 10.99 g/dL): treatment with IFA (100 mg iron, 500 µg folic acid) twice daily	
Gestational weight gain	
Weight monitoring every month; identification of inadequate weight gain (IWG) according to Institute of Medicine's guidelines	
Management of inadequate weight gain (IWG):	
- Nutritional Counselling	
- Hot meal (500 Kcal, 20 g protein) 6 days a week till delivery	
- Screening and treatment of infections (UTI, RTI, TB)	

**WaSH**<br>Provision of water filters, hand washing stations, water storage bottles, soap and disinfectants and counselling	
**PSYCHOSOCIAL SUPPORT**<br>Screen for depressive symptoms using PHQ-9 questionnaire<br>Counselling by study psychologist if PHQ score ≥10. Refer to psychiatrist if PHQ-9 score ≥15 and/or suicidal ideation.<br>Promotion of positive thinking and problem-solving skills<br>Screen for substance abuse and exposure to second-hand smoke and alcohol use in husbands and counsel	

*Composition given at the end

†Observed intake

Electronic monitoring system for tracking women with problems to support them for achieving intervention compliance across all domains.

EARLY CHILDHOOD

0-6 months: Mothers

Intervention group	Control Group‡
**HEALTH**<br>Facilitate hospital visit at 6 weeks postpartum and encourage compliance to supplements.	Routine postnatal and early childhood care
**NUTRITION**<br>Locally-prepared snacks daily and milk† 6 days a week (600 kcal, 20 g protein).<br>IFA (100 mg iron, 500 µg folic acid), Calcium (1000 mg) and vitamin D (400 IU) supplementation daily<br>Multiple micronutrients daily*	
**WaSH**<br>Continuation of all the WaSH interventions provided during pregnancy (water filters, water storage bottles, hand washing station, soap and disinfectants) and counselling on handwashing and hygiene practices (bathing the infant regularly, keeping the infant's surroundings clean, safe disposal of infant's feces, and handwashing before handling the baby).	
**PSYCHOSOCIAL CARE**<br>Promotion of positive thinking and problem-solving skills<br>Screening mothers for depressive symptoms and management as required<br>Counselling by study psychologist if PHQ score ≥10. Referral to psychiatrist if PHQ-9 score ≥15 and/or suicidal ideation.	

*Composition given at the end

†Observed intake

Electronic monitoring system for tracking women with problems to support them for achieving intervention compliance across all domains.

0-6 months: Infants

Intervention group	Control Group‡
**NUTRITION**<br>Initiation of breastfeeding (BF) within the first hour of birth.<br>Early lactation counselling for all mothers to prevent BF problems, resolution anytime during the first 6 months.<br>Counsel on exclusive BF till 6 months of age emphasizing exclusivity of BF from 3 up to 6 months of age.<br>Growth monitoring and management of IWG<br>Weight measurement on day 14 and thereafter monthly to identify IWG (<15th centile as per WHO Growth Velocity Standards, i.e. weight gain <20 g/day between ages day 14 to month 2; <15 g/d for months 3 and 4; <10 g/d for months 5 to 6) for all term infants. Management through: lactation counselling, screening and treatment of infections, facility based management of IWG by senior paediatrician at Safdarjung Hospital after 15 days of continued efforts of lactation support and no medical cause is identified<br>Additional support for LBW babies and preterm babies (even if not LBW). BF support by lactation counselling through home visits in the first three months (biweekly in first month, weekly in second and third month, monthly from fourth to sixth month).<br>Offer expressed breastmilk feeding for preterm babies only after a breastfeed.<br>Extended support for preterms (through assessment of feeding, growth and investigations within 4-6 weeks after birth) after discharge and ensuring that the advice given at the hospital is followed at home.<br>Support kangaroo mother care at home<br>Vitamin D 400 IU daily for all infants up to 6 months<br>Iron supplementation up to 6 months: VLBW from 2 weeks, LBW from 6 weeks	Routine postnatal and early childhood care
**HEALTH**<br>Educating mother and other family members to identify danger signs and in early care seeking for illness.<br>Facilitating referral to health facility for infants with any danger signs or illness requiring facility-based management.<br>Counselling on timely immunisation	
**PSYCHOSOCIAL CARE**<br>Counselling, demonstration and practice sessions for mothers at each home visit on early child play and responsive care.<br>Identification of delayed development and timely referral for further management	

Electronic monitoring system for tracking infants with problems to support them for achieving intervention compliance across all domains.

6-24 months: Children

Intervention group	Control‡
**NUTRITION**<br>Effective counselling on initiation of complementary feeding by preparing the mother and family 1-2 weeks prior to 6 months of infant's age<br>Initiation of complementary feeding at 6 months of age and teaching the mother by demonstrations on how to prepare foods at home which can be fed easily to the child 6	Routine early childhood care

months onwards.<br>Provision of daily food supplement with 125 kcal, 2.5 g protein up to 12 months and 250 kcal energy and 5 g protein from 12 to 24 months that includes 80 to 100% RDA of micronutrients<br>Counselling for intake of home-based food<br>Counselling and demonstration of responsive feeding to mother and family members<br>IFA (10 mg iron and 100 mcg folic acid) supplementation daily up to 24 months<br>Lactation counselling for supporting continued BF till 24 months of age<br>Growth monitoring (weight and length monthly); management of IWG (<25th centile as per WHO Growth Velocity Standards) by providing additional food supplements in form of snacks (125 kcal and 2.5 g protein up to 12 months, and 250 kcal energy and 5 g protein from 12 to 24 months) till child has adequate weight gain for 2 consecutive months; nutritional counselling; screening and treatment of infections<br>Home based management of moderate acute malnutrition through counselling, on preparation of augmented home-based foods.<br>Facilitating facility-based management of severe acute malnutrition.	
**HEALTH**<br>Educating mother and other family members to identify danger signs, and in early care seeking.<br>Facilitating medical management.<br>Counselling on feeding the child during and after illness.<br>Counselling on timely immunization<br>Provision of Albendazole (200 mg) for deworming starting 12 months of age and then 6 monthly	
**WaSH**<br>Continuation of all the WaSH interventions provided during pregnancy (water filters, water storage bottles, hand washing stations, soap, disinfectants) and counselling on hygiene practices (safe preparation of food, storage and feeding of the child utilizing clean utensils and clean water for cooking and drinking).<br>Clean play area for children; provide play mat.<br>Safe disposal of child's faeces; provide potty.	
**PSYCHOSOCIAL SUPPORT**<br>**CHILDREN**<br>Counselling on early child development.<br>Demonstration and practice session for mother at each home visit on early child play and responsive care.<br>Identification of delayed development three-monthly or as response to parental concerns and timely referral to developmental psychologist.<br>**MOTHERS**<br>Promotion of positive thinking and problem-solving skills.<br>Screening mothers for depressive symptoms. Counselling by study psychologist if PHQ score ≥10. Referral to psychiatrist if PHQ-9 score ≥15 and/or suicidal ideation.	

Electronic monitoring system for tracking children and mothers with problems to support them for achieving intervention compliance across all domains.

[‡]National Programs for women of reproductive age, pregnant women and undertwos (Table S2)

***Composition of multiple micronutrient tablet**

Micronutrient	per tablet	Micronutrient	per tablet
Vitamin A (mcg)	750	Zinc (mg)	15
Vitamin D (mcg)	5	Iodine (mcg)	150
Vitamin K (mcg)	10	Selenium (mcg)	25
Vitamin C (mg)	50	Magnesium (mg)	100
Vitamin E (mg a-TE)	10	Copper (mg)	2
Thiamine (mg)	2	Manganese (mg)	2.5
Riboflavin (mg)	3	Phosphorus (mg)	125
Niacin (mg)	26	Potassium (mg)	40
Pyridoxine (mg)	1.5	Phosphorus (mg)	125
Pantothenate (mg)	5	Chloride (mg)	36.3
Folate (mcg)	300	Chromium (mcg)	25
Vitamin B12 (mcg)	1	Molybdenum (mcg)	25
Biotin (mcg)	30	Nickel (mcg)	5
Iron (mg)	9	Silicon (mcg)	10
Calcium (mg)	162	Vanadium (mcg)	10

Table S3. Secondary outcomes in women and timing of measurement

**Outcomes**	**Time Points**						
	**End of pre-conception period or reporting of pregnancy**	**Gestational age 26-28 weeks**	**Gestational age 35-36 weeks**	**At birth or day 7**	**Month 2**	**Month 6**	**Month 12**
Birth interval	✓						
Body mass index	✓					✓	✓
Weight Gain		✓	✓				
Symptoms of reproductive tract infections	✓		✓				
Depressive symptoms	✓				✓		✓
Inflammatory markers (C-reactive protein, Alpha-acid glycoprotein) in a subsample	✓		✓			✓	
Micronutrient status (vitamin A, D, B12, zinc, iron, folate and selenium) in a subsample	✓		✓			✓	
Anemia status	✓		✓			✓	
Thyroid status	✓						
Diabetes status	✓						
Postpartum morbidity				✓			
Pregnancy outcomes, still birth				✓			

Secondary outcomes in children and timing of measurement

**Outcomes**	**Time points**										
	**At birth or day 7**	**Month 1**	**Month 3**	**Month 5**	**Month 6**	**Month 9**	**Month 12**	**Month 15**	**Month 18**	**Month 21**	**Month 24**
Attained length (length-for- age z score)					✓		✓				
Attained weight (weight-for-age z score)					✓		✓				✓
Stunted (length-for- age <-2 SD of the WHO Child Growth Standards)					✓		✓				
Underweight (weight-for-age z score <−2 SD of the WHO Child Growth Standards)					✓		✓				✓
Wasted (weight-for-height z score <−2 SD of the WHO Child Growth Standards)					✓		✓				✓
Weight and length trajectories	✓	✓	✓	✓	✓	✓	✓	✓	✓	✓	✓
Head circumference	✓						✓				✓
Mid-upper arm circumference					✓		✓				✓
Body composition in a sub sample		✓									
Caregiver reported development outcomes							✓				✓

Outcomes		Time points									
	At birth or day 7	Month 1	Month 3	Month 5	Month 6	Month 9	Month 12	Month 15	Month 18	Month 21	Month 24
in a sub sample											
Cognitive, language and motor scores in a sub sample											✓
Mother-infant bonding in a sub sample					✓		✓		✓		
Micronutrient status (vitamin A, D, B12, zinc, iron, folate and selenium) in a subsample											✓
Anemia status											✓
Inflammatory markers: C-reactive protein, Alpha acid glycoprotein in a sub sample											✓
Morbidity: Severe infection in neonatal period, 2 weeks prevalence of diarrhea, pneumonia, dysentery		✓	✓		✓		✓		✓		✓
Hospitalizations		✓	✓		✓		✓		✓		✓
Early initiation of breastfeeding	✓										
Exclusive breastfeeding		✓		✓							
Continued breastfeeding							✓		✓		✓
Complementary feeding: 24 dietary recalls in a sub sample						✓	✓		✓		✓

Figure S1. Subgroup analysis for low birth weight comparing Preconception interventions and No preconception interventions (Group A+B vs Group C+D)

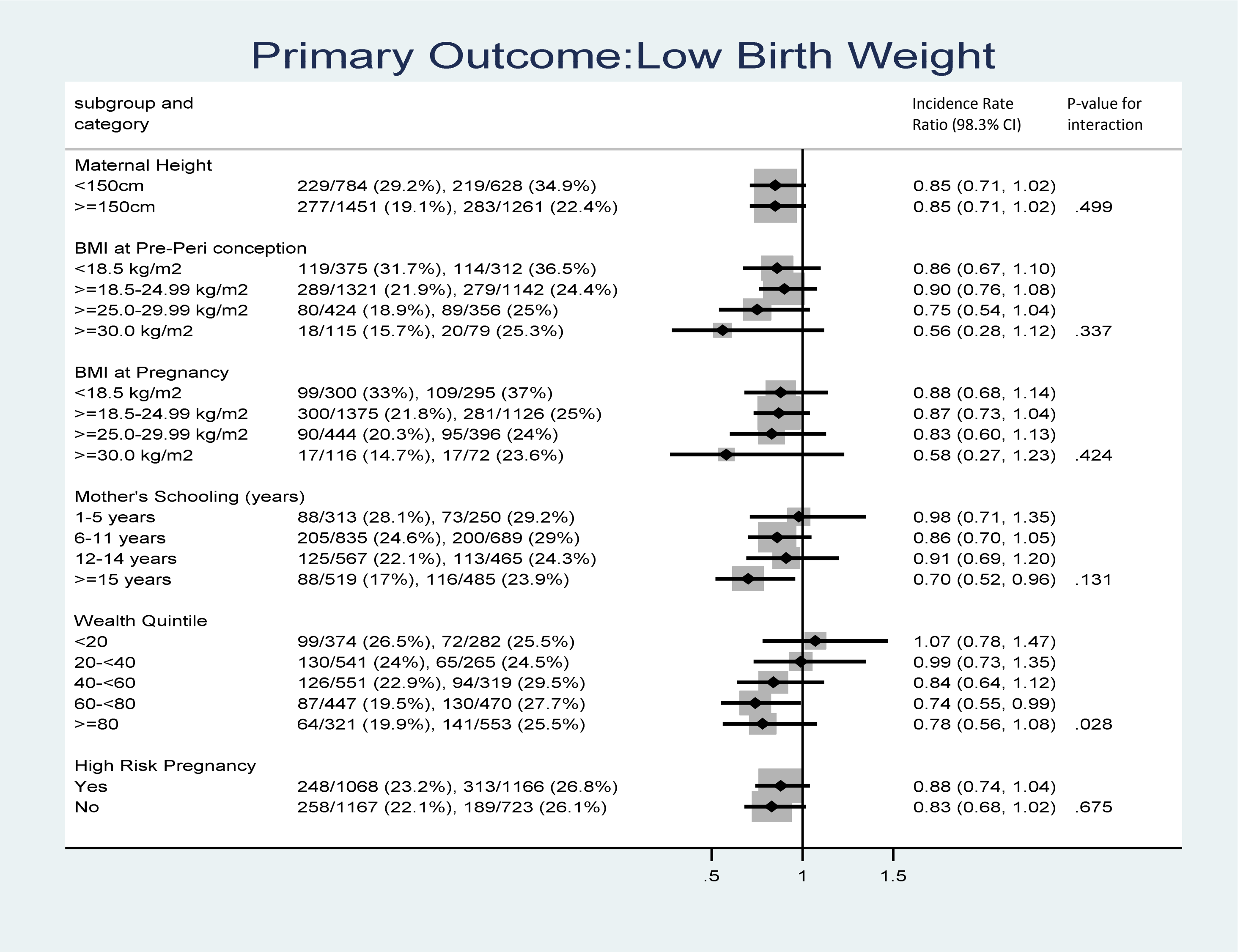

Figure S2. Subgroup analysis for low birth weight comparing Pregnancy and early childhood interventions and No pregnancy and early childhood interventions (Group A+C vs Group B+D)

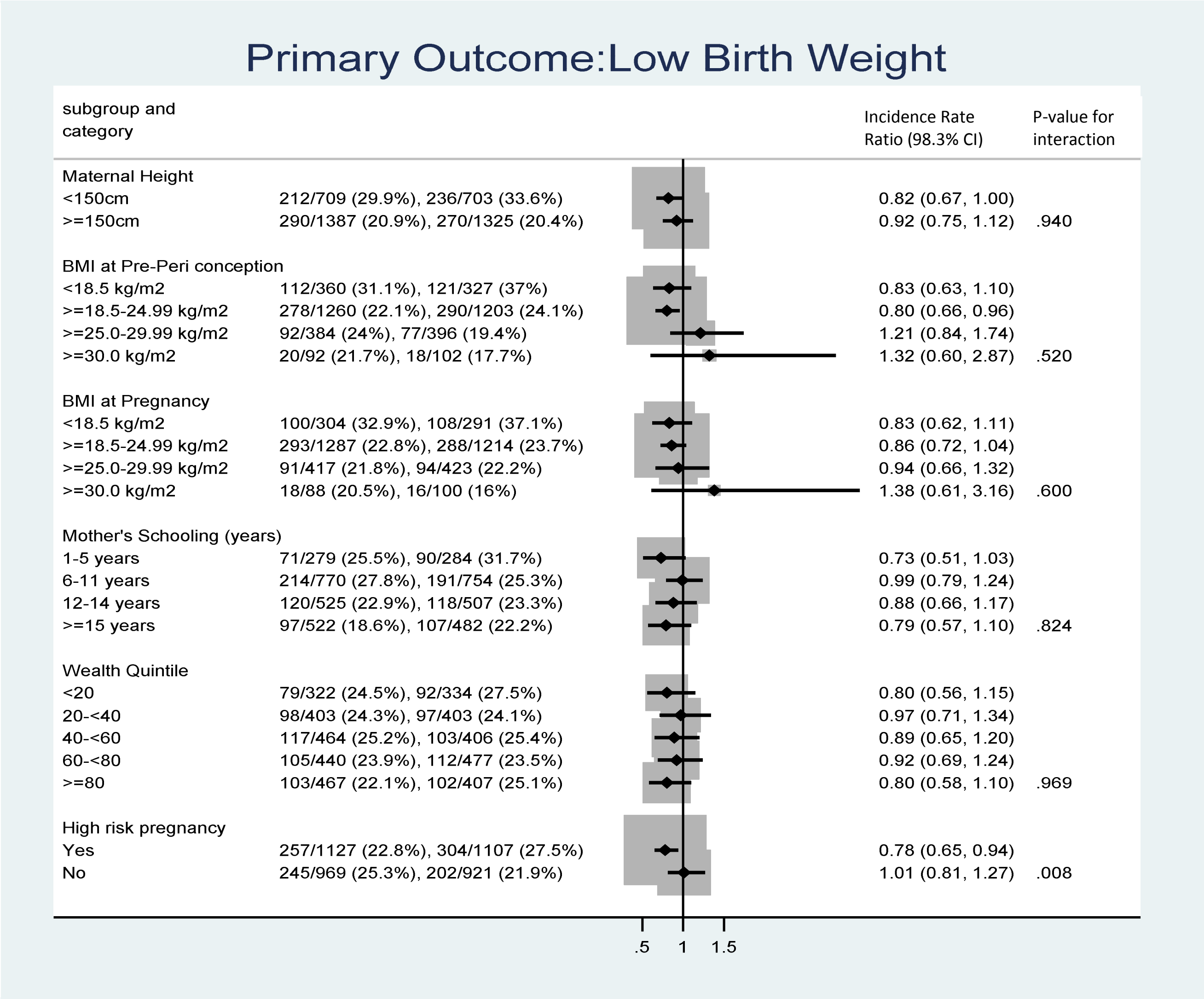

Figure S3. Subgroup analysis for low birth weight comparing Preconception and pregnancy and early childhood interventions and No preconception and pregnancy and early childhood interventions (Group A vs Group D)

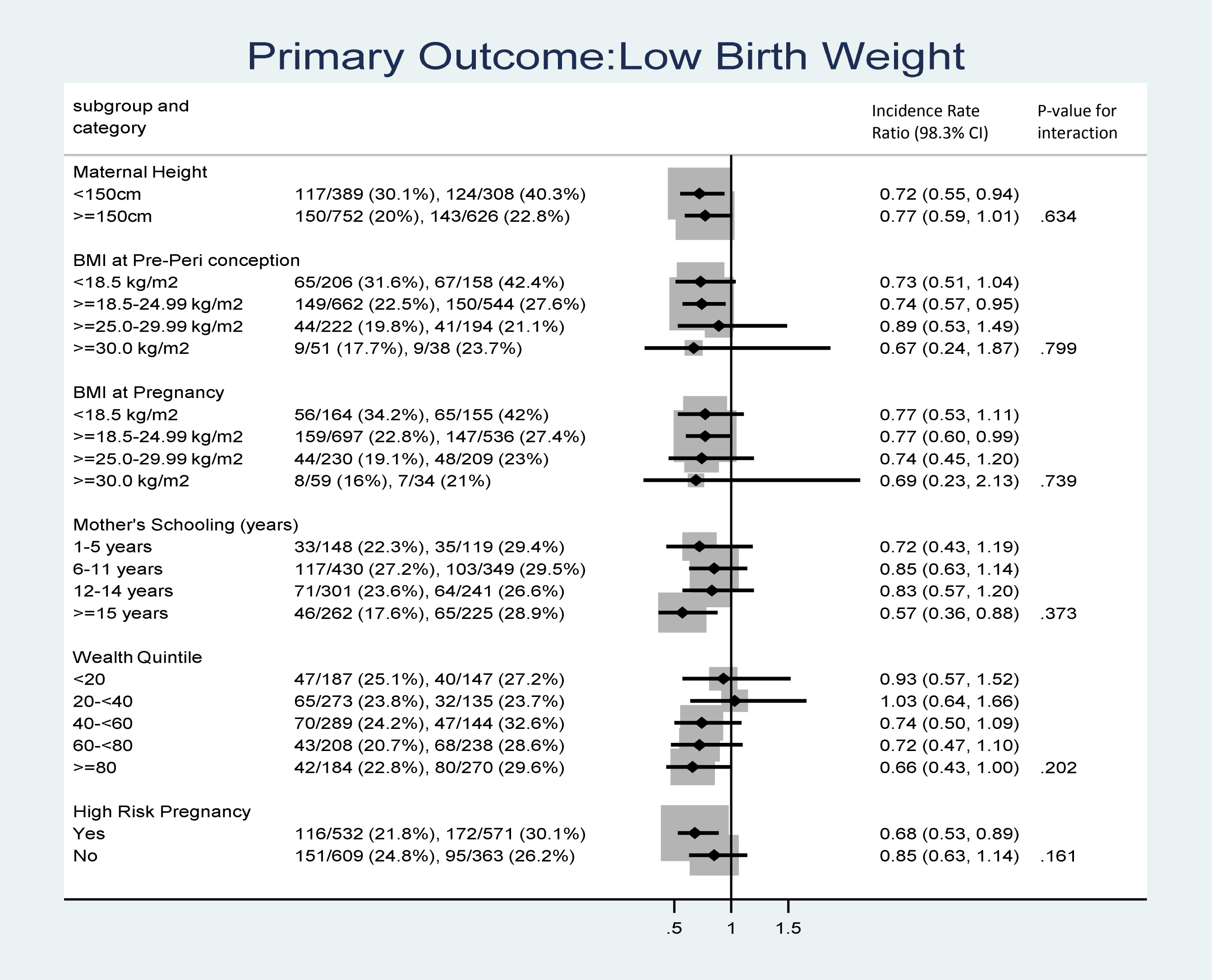

Figure S4. Subgroup analysis for stunting at 24 months comparing Preconception interventions and No preconception interventions (Group A+B vs Group C+D)

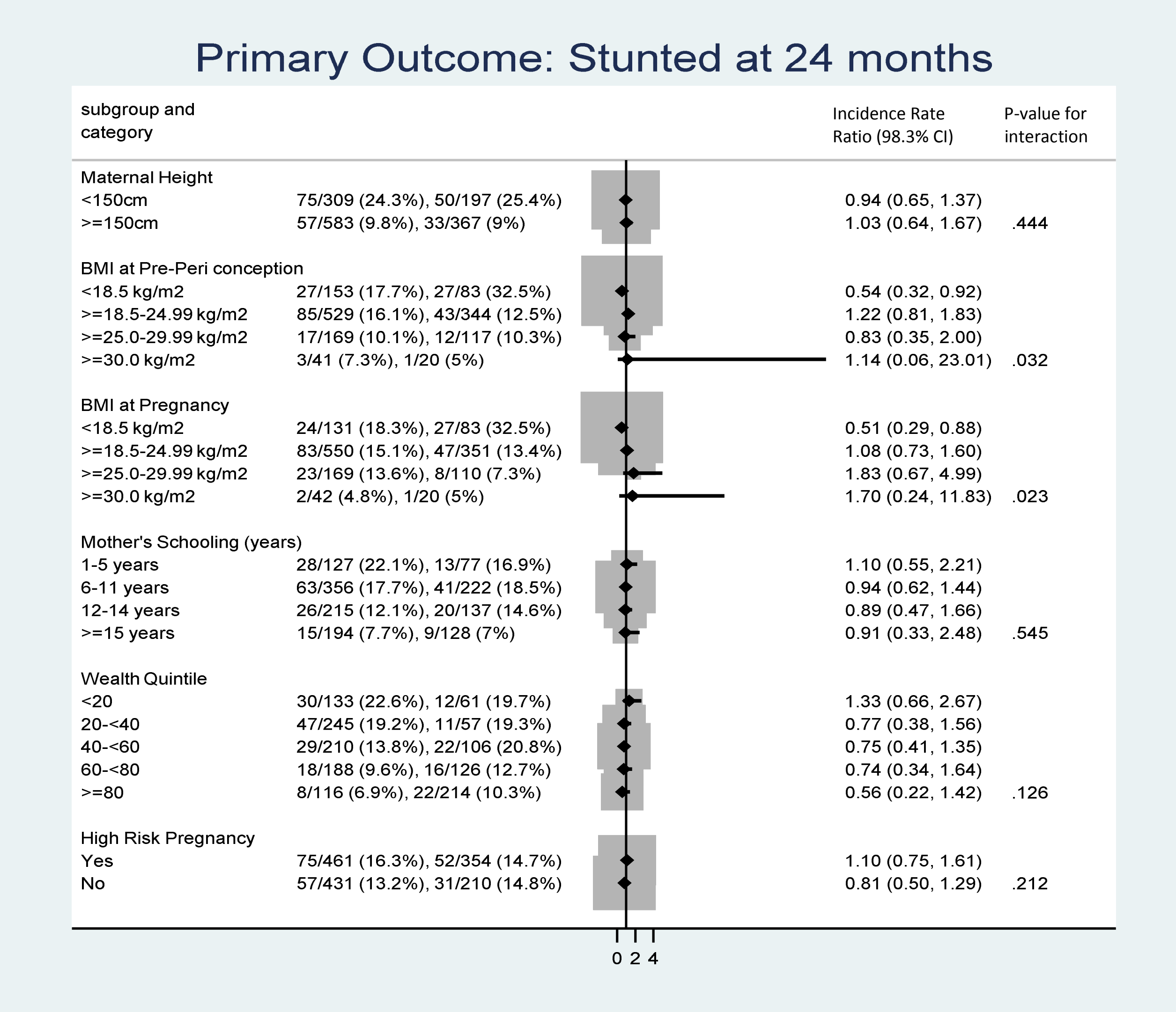

Figure S5. Subgroup analysis for stunting at 24 months comparing Pregnancy and early childhood interventions and No pregnancy and early childhood interventions (Group A+C vs Group B+D)

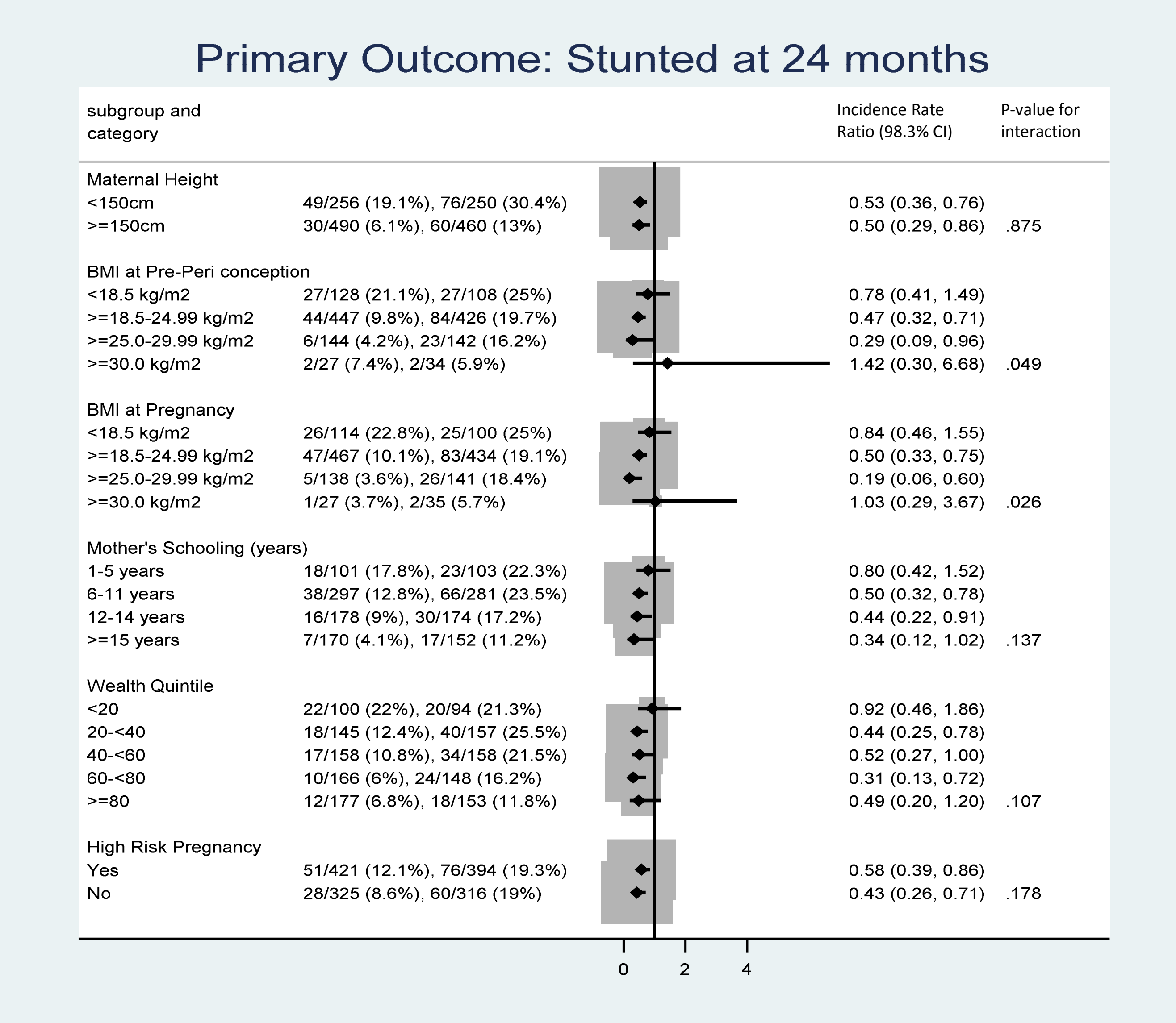

Figure S6. Subgroup analysis for stunting at 24 months comparing Preconception and pregnancy and early childhood interventions and No preconception and pregnancy and early childhood interventions (Group A vs Group D)

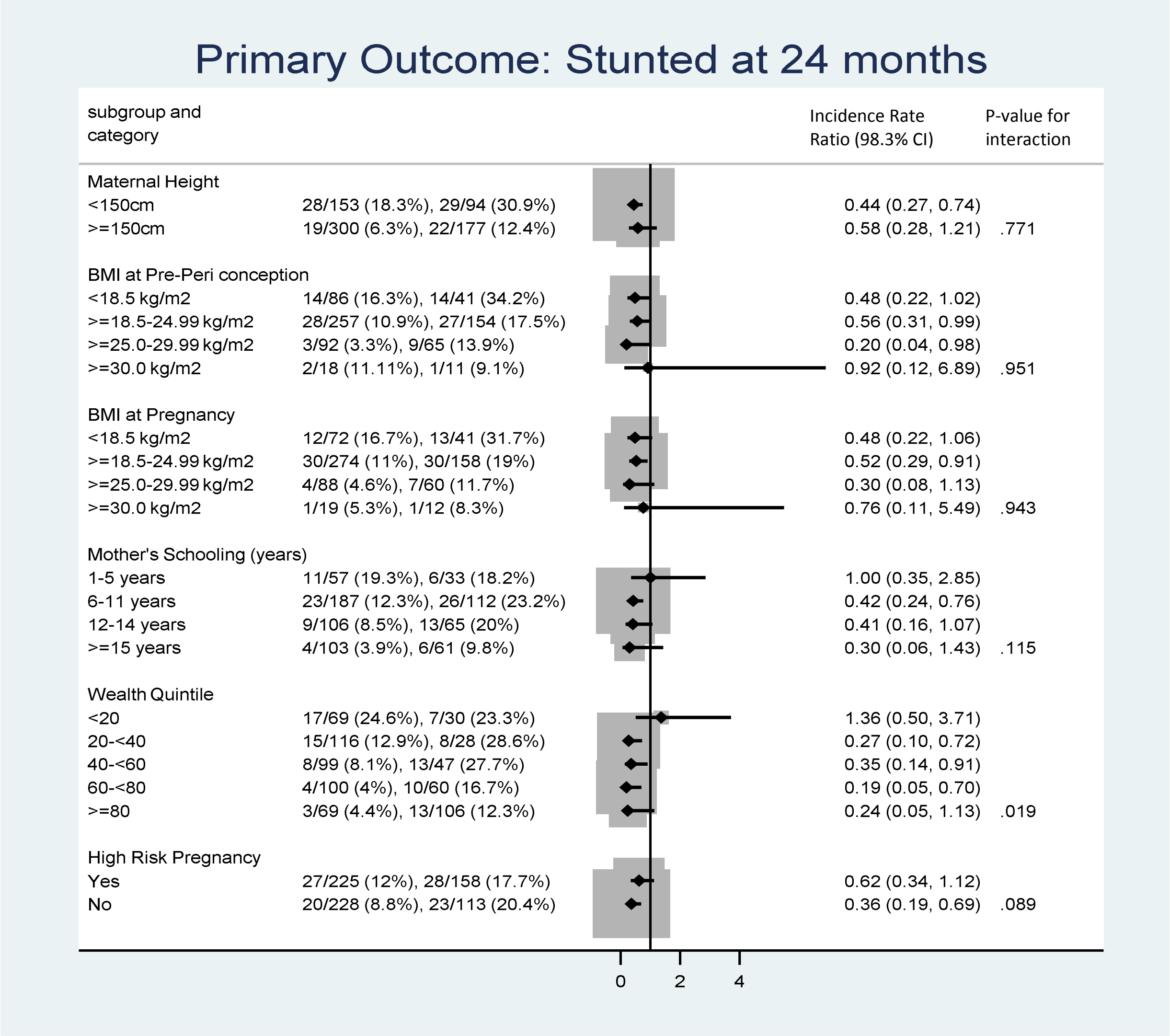

Figure S7. Weight-for-age z-scores from birth to 24 months; cross-sectional data

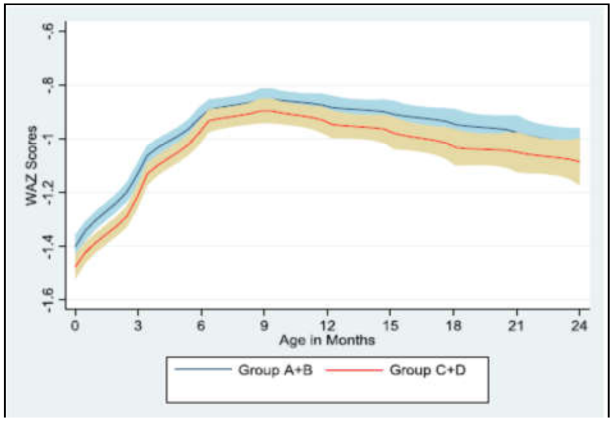

Shaded area 95% CI
Numbers at each age: Group A+B - 2235 at 0 mo, 2219 at 3 mo, 2259 at 6 mo, 2058 at 9 mo, 1874 at 12 mo, 1563 at 15 mo, 1298 at 18 mo, 1061 at 21 mo, 892 at 24 mo; Group C+D - 1889 at 0 mo, 1860 at 3 mo, 1868 at 6 mo, 1624 at 9 mo, 1409 at 12 mo, 1128 at 15 mo, 887 at 18 mo, 709 at 21 mo, 564 at 24 mo

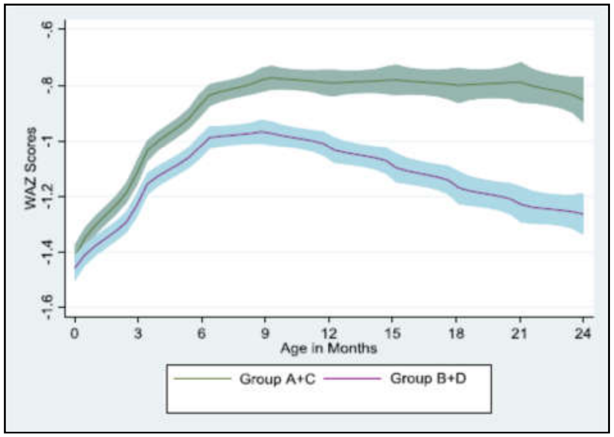

Shaded area 95% CI
Numbers at each age: Group A+C - 2096 at 0 mo, 2077 at 3 mo, 2113 at 6 mo, 1888 at 9 mo, 1675 at 12 mo, 1360 at 15 mo, 1107 at 18 mo, 903 at 21 mo, 746 at 24 mo; Group B+D - 2028 at 0 mo, 2002 at 3 mo, 2014 at 6 mo, 1794 at 9 mo, 1608 at 12 mo, 1331 at 15 mo, 1078 at 18 mo, 867 at 21 mo, 710 at 24 mo

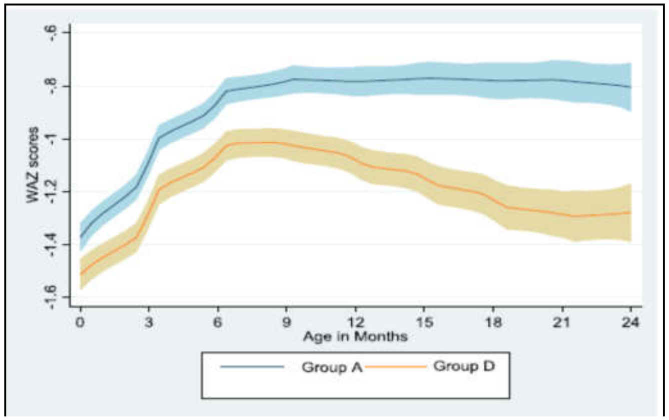

Shaded area 95% CI
Numbers at each age: Group A - 1141 at 0 mo, 1137 at 3 mo, 1160 at 6 mo, 1053 at 9 mo, 953 at 12 mo, 787 at 15 mo, 658 at 18 mo, 540 at 21 mo, 453 at 24 mo; Group D - 934 at 0 mo, 920 at 3 mo, 915 at 6 mo, 789 at 9 mo, 687 at 12 mo, 555 at 15 mo, 438 at 18 mo, 346 at 21 mo, 271 at 24 mo

Group A+B vs Group C+D: Effect of preconception interventions: women who received interventions compared with those who did not receive preconception interventions; **Group A+C vs Group B+D**: Effect of pregnancy and early childhood interventions: women who received these interventions compared with those who did not receive these interventions; **Group A vs Group D:** Effect of preconception, pregnancy and early childhood interventions: women who received interventions in these periods compared with those who received routine care

Table S4. Sensitivity analysis for primary outcomes at birth and 24 months

Outcomes	Group A+B vs Group C+D: Preconception interventions			Group A+C vs Group B+D: Pregnancy and early childhood interventions			Group A vs Group D: Preconception and pregnancy interventions and early childhood interventions		
	A+B n=2566	C+D n=2186	IRR or Mean difference (Holm Bonferroni corrected CI and P values)	A+C n=2383	B+D n=2369	IRR or Mean difference (Holm Bonferroni corrected CI and P values)	A n=1290	D n=1093	IRR or Mean difference (Holm Bonferroni corrected CI and P values)
**At birth**									
Low birth weight*- n/total (%)†	506/2235 (22.6)	502/1889 (26.6)	0.85 (0.73 to 1.00) p=0.043**	502/2096 (24.0)	506/2028 (25.0)	0.87 (0.74 to 1.03) p=0.17	267/1141 (23.4)	267/934 (28.6)	0.76 (0.60 to 0.95) p=0.006**
Preterm birth - n/total (%)†	329/2542 (12.9)	270/2161 (12.5)	1.05 (0.84 to 1.31) p=1.00	281/2363 (11.9)	318/2340 (13.6)	0.85 (0.67 to 1.09) p=1.00	171/1283 (13.3)	160/1081 (14.8)	0.91 (0.66 to 1.25) P=1.0
Small-for-gestational age - n/total (%)†	643/2226 (28.9)	615/1870 (32.9)	0.87 (0.77 to 1.00) p=0.042**	589/2083 (28.3)	669/2013 (33.2)	0.80 (0.69 to 0.93) p=0.0001**	318/1138 (27.9)	344/925 (37.2)	0.71 (0.58 to 0.87) p<0.0001**
Mean (SD) Birth weight* - g‡	n=2235 2806 (443)	n=1889 2765 (449)	40.84 (0.82 to 80.9) p=0.041**	n=2096 2792 (438)	n=2028 2783 (455)	35.97 (-6.54 to 78.48) p=0.14	n=1141 2797 (438)	n=934 2744 (459)	77.67 (14.52 to 140.82) p=0.005**
Mean (SD) Birth length§ - cm‡	n=2263 48.1 (2.1)	n=1917 47.9 (2.2)	0.17 (-0.01 to 0.35) p=0.087	n=2138 48.0 (2.1)	n=2042 48.0 (2.1)	0.05 (-0.15 to 0.25) p=1.00	n=1161 48.0 (2.1)	n=940 47.9 (2.2)	0.21 (-0.07 to 0.49) p=0.22
**At 24 mo**									
Mean (SD) Length-for-age z score‡	-0.8 (1.2)	-0.8 (1.1)	0.08 (-0.08 to 0.23) p=0.68	-0.6 (1.1)	-1.0 (1.1)	0.40 (0.23 to 0.57) p <0.0001**	-0.6 (1.2)	-1.0 (1.2)	0.46 (0.22 to 0.71) p<0.0001**
Stunted - n (%)†	132 (14.8)	83 (14.7)	0.96 (0.67 to 1.36) p=1.00	79 (10.6)	136 (19.2)	0.51 (0.35 to 0.76) p<0.0001**	47 (10.4)	51 (18.8)	0.49 (0.29 to 0.83) p=0.0011**

*Weight on day 7 (up to +6 days); †Adjusted incidence rate ratio (IRR); ‡Adjusted mean difference, adjusted for place of birth, family possesses below poverty line card, woman's height, woman BMI as potential confounders and twins for clustering within a household; §Length on days 1-7 (up to +6 days); **Significant (P values calculated using Holm-Bonferroni correction for multiple comparisons)

Stunted: Length-for-*age z score* <-2 SD of the WHO Child Growth Standards
Group A: Preconception, pregnancy and early childhood intervention; **Group B**: Only preconception intervention; **Group C**: Only pregnancy and early childhood intervention; **Group D**: Control

Group A+B vs Group C+D: Effect of preconception interventions: women who received interventions compared with those who did not receive preconception interventions ; **Group A+C vs Group B+D**: Effect of pregnancy and early childhood interventions: women who received these interventions compared with those who did not receive these interventions ; **Group A vs Group D:** Effect of preconception, pregnancy and early childhood interventions: women who received interventions in these periods compared with those who received routine care.

Table S5. Compliance to interventions during the preconception period

	At Enrollment		At 12 mo
**SCREENING AND TREATMENT**			
**HEALTH**			
Hypothyroidism			
Proportion screened (TSH) - n/total (%)	6672/6722 (99.3)		3083/3881 (79.4)
Proportion with hypothyroidism prescribed treatment - n/total (%)	851/1002 (84.9)		209/261 (80.1)
Pre-diabetes or diabetes			
Proportion screened (HbA1c) - n/total (%)	6672/6722 (99.3)		3080/3881 (79.4)
Proportion with pre-diabetes or diabetes prescribed treatment - n/total (%)	577/745 (77.5)		145/234 (62.0)
Hypertension			
Proportion screened (blood pressure) - n/total (%)	6680/6722 (99.4)		3257/3881 (83.9)
Proportion with hypertension prescribed treatment - n/total (%)	23/25 (92)		1/2 (50)
Reproductive tract infection (RTI)			
Proportion screened (symptoms) - n/total (%)	6683/6722 (99.4)		3316/3881 (85.4)
Proportion with RTI treated by physician - n/total (%)	966/1674 (57.7)		224/301 (74.4)
**NUTRITION**			
Anemia			
Proportion screened - n/total (%)	6673/6722 (99.3)		3079/3881 (79.3)
Median (IQR) percent days iron consumed by anemic women, n=3767, 1176	76 (53.6 to 92.5)		53.6 (25.0 to 75.0)
**PREVENTIVE AND PROMOTIVE INTERVENTIONS**	**Enrollment to 6 mo**	**6 to 12 mo**	**12 to 18 mo**
**NUTRITION**			
Median (IQR) percent weeks ferrous fumarate plus folic acid consumed by women (anemia prophylaxis), n=6668, 4989, 3820	88.9 (71.4 to 100)	86.7 (60 to 100)	85.7(50 to 100)
Median (IQR) percent days multiple micronutrients consumed, n=6638, 4887, 3765	66.7 (50 to 80.8)	70.8 (46.7 to 85.7)	66.7 (33.3 to 81.0)
Median (IQR) percent days egg or milk consumed by women with BMI <21 kg/m2, n=2791, 1547, 939	92 (80.8 to 95.5)	90.6 (79.3 to 95.7)	93.4 (83.3 to 97.0)
Median (IQR) percent days snacks consumed by women with BMI <18.5 kg/m2, n=1224, 563, 343	73.2 (50 to 87.2)	69.4 (50 to 80.4)	71.4 (49.0 to 83.3)
**PSYCHOSOCIAL INTERVENTION**			
Proportion screened for depressive symptoms (PHQ-9)	12246/12770 (95.9)	8316/9411 (88.4)	6130/7331 (83.6)
Proportion with depressive symptoms (PHQ-9 ≥10 or suicidal ideation) initiated treatment	138/208 (66.3)	49/99 (49.5)	20/33 (60.6)
Proportion counselled for positive thinking and problem solving skills	12246/12770 (95.9)	8316/9411 (88.4)	6130/7331 (83.6)
**WATER, SANITATION AND HYGIENE (WaSH)**			
Proportion of women received WaSH counselling	12111/12770 (94.8)	8189/9411 (87.0)	6059/7331 (82.6)

Anemia: Hb <12 g/dL, Hypothyroidism: TSH levels were >5.5 mIU/L or if TSH levels were between 4.0 and 5.5 mIU/L and Anti-TPO Ab positive, Prediabetes: HbA1c between 5.7% and 6.4%, Hypertension: systolic blood pressure ≥140 mmHg and/or diastolic blood pressure ≥90 mmHg, Reproductive tract infection: Any symptoms of vaginal discharge, itching, burning, swelling, ulcer in genital region, swelling and pain lower abdomen

Table S6. Compliance to interventions during pregnancy

**HEALTH**	
**Anemia**	
Proportion screened - n/total (%)	2359/2460 (95.9)
Proportion received treatment - n/total (%)	1305/1334 (97.8)
**Urinary tract infections**	
Proportion screened - n/total (%)	2191/2460 (89.1)
Proportion provided treatment - n/total (%)	146/180 (81.1)
**Reproductive tract infections**	
Proportion screened (symptoms) - n/total (%)	2435/2460 (99.0)
Proportion treated by physician - n/total (%)	436/439 (99.3)
**Gestational Diabetes Mellitus**	
Proportion screened - n/total (%)	2132/2460 (86.7)
Proportion provided treatment - n/total (%)	125/128 (97.6)
**Hypothyroidism**	
Proportion screened - n/total (%)	2297/2460 (93.4)
Proportion prescribed treatment - n/total (%)	176/181 (97.2)
**Hypertension**	
Proportion screened (blood pressure) - n/total (%)	1980/2460 (80.5)
Proportion prescribed treatment - n/total (%)	5/7 (71.4)
**NUTRITION**	
Median (IQR) percent days micronutrient supplement consumed, n=2460	81.8 (71.4 to 88.9)
Median (IQR) percent days iron-folic acid consumed n=2460	83.3 (75.0 to 89.6)
Median (IQR) percent days calcium and Vitamin D supplement consumed, n=2460	81.7 (72.7 to 88.1)
Median (IQR) percent days snacks consumed by women with BMI <25 kg/m^2, n=1865	73.0 (58.3 to 82.9)
Median (IQR) percent days egg or milk consumed by women with BMI <30 kg/m^2, n=2355	83.3 (71.4 to 90.5)
**PSYCHOSOCIAL INTERVENTION**	
Proportion screened for depressive symptoms (PHQ-9) - n/total (%)	2381//2460 (96.8)
Proportion women received counselling on positive thinking and problem solving skills - n/total (%)	2381//2460 (96.8)
**WATER, SANITATION AND HYGIENE (WaSH)**	
Proportion women who provided water filter - n/total (%)	1878/2460 (76.3)*
Proportion homes where hand washing stations were installed - n/total (%)	1010/2460 (41.1)*

Anemia: Hb <11 g/dL, Urinary tract infection: Urine culture microbial growth of 10^5 CFU/ml, Reproductive tract infection: Any symptoms of vaginal discharge, itching, burning, swelling, ulcer in genital region, swelling and pain lower abdomen, Gestational Diabetes mellitus: 2 hr blood sugar ≥140 mg/dL in Oral Glucose Tolerance Test, Hypothyroidism: TSH levels were >2.6 mIU/L, Hypertension: Systolic blood pressure ≥140 mmHg and/or diastolic blood pressure ≥90 mmHg

*Remaining households already had a water filter and handwashing station at home

Table S7. Compliance to interventions in mothers during the postnatal period

INTERVENTION	
**NUTRITION**	n=2382
Median (IQR) percent days micronutrient supplements consumed	74.3 (48.6 to 91.4)
Median (IQR) percent days iron-folic acid consumed	74.3 (48.6 to 91.4)
Median (IQR) percent days calcium and vitamin D supplement consumed	68.6 (45.7 to 88.6)
Median (IQR) percent snacks consumed	80 (54.3 to 94.3)
Median (IQR) percent days milk consumed	82.7 (61.5 to 92.6)
**PSYCHOSOCIAL**	
Women screened for depressive symptoms (PHQ-9), n (%)	2370 (99.5)
Women who received counselling on positive thinking and problem-solving skills, n (%)	2370 (99.5)

Table S8. 24-hour dietary recalls in a randomly selected subset of women during preconception, pregnancy and postpartum periods

	Preconception Interventions	No Preconception Interventions	Mean difference (98.3% CI)
**Preconception period**	**n=58**	**n=63**	
Mean (SD) Energy, kcal	1772 (624)	1468 (474)	330 (90 to 570)
Mean (SD) Protein, g	56 (22)	45 (15)	12 (4 to 21)
	**Pregnancy and early childhood interventions**	**No pregnancy and early childhood interventions**	
**Pregnancy period**	**n=102**	**n=104**	
Mean (SD) Energy, kcal	2085 (748)	1626 (529)	429 (199 to 660)
Mean (SD) Protein, g	67 (26)	50 (18)	16 (8 to 24)
**Postpartum period at infant age 3 months**	**n=111**	**n=90**	
Mean (SD) Energy, kcal	2287 (596)	1648 (491)	686 (461 to 911)
Mean (SD) Protein, g	75 (20)	50 (16)	27 (20 to 34)

* Adjusted mean difference 98.3% CI), adjusted for place of birth, family possesses below poverty line card, woman's height, woman BMI as potential confounders and twins for clustering within a household

Table S9. Compliance to interventions in children from birth to 24 months

Interventions	n/N (%)
Received counselling for danger signs - n/total (%)	740/746 (99.2)
**Birth to 6 months**	
Received lactation counselling	740/746 (99.2)
Median (IQR) percent days vitamin D consumed, n=746	94.3 (80 to 100)
Median (IQR) days Iron consumed (for low birth weight), n=168	64.3 (51.4 to 80)
**6 to 24 months**	
Median (IQR) days iron consumed, n=746	75.7 (61.4 to 84.3)
Median (IQR) days milk cereal mix consumed, n=746	68.6 (52.9 to 80)
Received early child play and responsive care - n/total (%)	735/746 (98.5)
Received play mats - n/total (%)	742/746 (99.5)
Received potty - n/total (%)	742/746 (99.5)

Table S10. Secondary outcomes: Anthropometry at 6 and 12 months of age

Outcomes	Groups				Preconception intervention groups vs no preconception intervention groups A+B vs C+D		Pregnancy intervention groups vs no pregnancy intervention group A+C vs B+D		Preconception and pregnancy intervention group vs no preconception and pregnancy intervention group A vs D	
	A	B	C	D	IRR (98.3%)	ARR (%) or MD (98.3%)	IRR (98.3%)	ARR (%) or MD (98.3%)	IRR (98.3%)	ARR (%) or MD (98.3%)
**Infant age 6 mo**	**n=1160**	**n=1099**	**n=953**	**n=914**						
Mean (SD) length-for-age z score*	-0.7 (1.1)	-0.7 (1.1)	-0.7 (1.1)	-0.8 (1.1)	-	0.03 (-0.05 to 0.11)	-	0.10 (0.02 to 0.19)	-	0.13 (0.01 to 0.25)
Stunted, n (%)†	121 (10.4)	124 (11.3)	99 (10.4)	115 (12.6)	0.93 (0.76 to 1.15)	-0.18 (-2.28 to 1.92)	0.85 (0.68 to 1.07)	-1.61 (-3.78 to 0.56)	0.82 (0.60 to 1.13)	-1.66 (-5.03 to 1.71)
Mean (SD) weight for length z score*	-0.5 (1.1)	-0.6 (1.0)	-0.5 (1.1)	-0.6 (1.1)	-	0.03 (-0.05 to 0.11)	-	0.16 (0.07 to 0.24)	-	0.18 (0.06 to 0.31)
Wasted, n (%)†	94 (8.1)	94 (8.6)	76 (8.0)	96 (10.5)	0.91 (0.71 to 1.16)	-0.58 (-2.67 to 1.51)	0.79 (0.61 to 1.02)	-2.29 (-4.52 to -0.06)	0.75 (0.53 to 1.06)	-2.62 (-6.00 to 0.76)
Mean (SD) weight for age z score*	-0.8 (1.1)	-1.0 (1.1)	-0.9 (1.1)	-1.0 (1.1)	-	0.04 (-0.04 to 0.12)	-	0.18 (0.10 to 0.27)	-	0.22 (0.10 to 0.34)
Underweight, n (%)†	145 (12.5)	194 (17.7)	133 (14.0)	150 (16.4)	0.99 (0.83 to 1.18)	0.84 (-1.66 to 3.34)	0.74 (0.61 to 0.89)	-4.23 (-6.83 to -1.62)	0.74 (0.57 to 0.98)	-3.04 (-6.74 to 0.66)
Mean (SD) MUAC, cm*	13.9 (1.1)	13.7 (1.0)	13.9 (1.1)	13.6 (1.0)	-	0.03 (-0.05 to 0.11)	-	0.22 (0.14 to 0.31)	-	0.24 (0.12 to 0.36)
Mean (SD) Head circumference, cm*	41.3 (1.4)	41.2 (1.4)	41.3 (1.4)	41.2 (1.5)	-	0.01 (-0.09 to 0.12)	-	0.18 (0.06 to 0.29)	-	0.18 (0.01 to 0.34)
**Infant age 12 mo**	**n=953**	**n=921**	**n=722**	**n=687**						
Mean (SD) length-for-age z score*	-0.8 (1.1)	-0.9 (1.1)	-0.8 (1.1)	-1.0 (1.1)	-	0.04 (-0.05 to 0.13)	-	0.19 (0.10 to 0.28)	-	0.24 (0.11 to 0.37)
Stunted, n (%)†	115 (12.1)	160 (17.4)	100 (13.9)	111 (16.2)	0.98 (0.81 to 1.19)	0.08 (-2.59 to 2.75)	0.75 (0.61 to 0.92)	-2.91 (-5.68 to -0.14)	0.74 (0.54 to 1.00)	-2.85 (-6.85 to 1.14)
Mean (SD) weight for length z score*	-0.6 (1.0)	-0.7 (1.0)	-0.6 (1.1)	-0.8 (1.0)	-	0.05 (-0.04 to 0.13)	-	0.22 (0.13 to 0.31)	-	0.30 (0.17 to 0.43)
Wasted, n (%)†	74 (7.8)	90 (9.8)	52 (7.2)	78 (11.4)	0.94 (0.72 to 1.23)	-0.15 (-2.45 to 2.15)	0.70 (0.53 to 0.93)	-3.50 (-5.89 to -1.11)	0.64 (0.44 to 0.93)	-4.30 (-7.95 to -0.66)
Mean (SD) weight for age z score*	-0.8 (1.1)	-1.0 (1.1)	-0.8 (1.1)	-1.1 (1.1)	-	0.05 (-0.03 to 0.14)	-	0.26 (0.17 to 0.35)	-	0.34 (0.21 to 0.47)
Underweight, n (%)†	121 (12.7)	152 (16.5)	88 (12.2)	123 (17.9)	0.97 (0.80 to 1.19)	0.64 (-2.13 to 3.42)	0.71 (0.58 to 0.88)	-4.38 (-7.27 to -1.48)	0.67 (0.51 to 0.89)	-5.76 (-10.04 to -1.48)

Outcomes	Groups				Preconception intervention groups vs no preconception intervention groups A+B vs C+D		Pregnancy intervention groups vs no pregnancy intervention group A+C vs B+D		Preconception and pregnancy intervention group vs no preconception and pregnancy intervention group A vs D	
	A	B	C	D	IRR (98.3%)	ARR (%) or MD (98.3%)	IRR (98.3%)	ARR (%) or MD (98.3%)	IRR (98.3%)	ARR (%) or MD (98.3%)
Mean (SD) MUAC*, cm*	14.2 (1.1)	14.1 (1.1)	14.2 (1.1)	14.0 (1.1)	-	0.03 (-0.06 to 0.12)	-	0.24 (0.15 to 0.34)	-	0.31 (0.18 to 0.44)
Mean (SD) Head circumference*, cm*	43. 9 (1.5)	43.8 (1.4)	43.9 (1.4)	43.7 (1.4)	-	0.04 (-0.08 to 0.15)	-	0.19 (0.07 to 0.31)		0.22 (0.05 to 0.39)

LAZ: length-for-age z score, WLZ: weight- for-length z score, WAZ: weight- for-age z score

*Adjusted mean difference (98.3% CI), †Adjusted Incidence Rate Ratio (IRR), 98.3% CI, adjusted for place of birth, family possesses below poverty line card, woman's height, woman BMI as potential confounders and twins for clustering within a household. Not corrected for multiple outcomes or comparisons.

Group A: Preconception, pregnancy and early childhood intervention; **Group B**: Only preconception intervention; **Group C**: Only pregnancy and early childhood intervention; **Group D**: Control

Group A+B vs Group C+D: Effect of preconception interventions: women who received interventions compared with those who did not receive preconception interventions ; **Group A+C vs Group B+D**: Effect of pregnancy and early childhood interventions: women who received these interventions compared with those who did not receive these interventions ; **Group A vs Group D:** Effect of preconception, pregnancy and early childhood interventions: women who received interventions in these periods compared with those who received routine care.

Table S11. Secondary outcomes: Breastfeeding at infant ages 1, 5 and 12 months

Outcomes	Groups				Preconception intervention groups vs no preconception intervention groups A+B vs C+D		Pregnancy intervention groups vs no pregnancy intervention group A+C vs B+D		Preconception and pregnancy intervention group vs no preconception and pregnancy intervention group A vs D	
	**A**	**B**	**C**	**D**	**IRR (98.3%)**	**ARR (%) or MD (98.3%)**	**IRR (98.3%)**	**ARR (%) or MD (98.3%)**	**IRR (98.3%)**	**ARR (%) or MD (98.3%)**
**Exclusive breastfeeding**										
1 month – n/total (%)*	844/1110 (76.0)	583/1073 (54.3)	748/983 (76.1)	528/950 (55.6)	0.99 (0.94 to 1.05)	-0.43 (-4.04 to 3.18)	1.37 (1.29 to 1.45)	20.45 (16.63 to 24.26)	1.36 (1.25 to 1.48)	20.24 (14.86 to 25.62)
5 months – n/total (%)*	885/1219 (72.6)	296/1203 (24.6)	770/1023 (75.3)	289/1002 (28.8)	0.93 (0.87 to 1.00)	-3.87 (-7.53 to -0.22)	2.78 (2.54 to 3.05)	47.68 (44.20 to 51.15)	2.57 (2.25 to 2.92)	45.15 (40.11 to 50.19)
**Continued breastfeeding**										
12 months – n/total (%)*	863/948 (91.0)	767/919 (83.5)	660/718 (91.9)	565/683 (82.7)	1.00 (0.97 to 1.03)	-0.08 (-2.93 to 2.77)	1.10 (1.06 to 1.14)	8.76 (5.61 to 11.91)	1.10 (1.05 to 1.16)	8.76 (4.12 to 13.41)

*Adjusted Incidence Rate Ratio (IRR), 98.3% CI, adjusted for place of birth, family possesses below poverty line card, woman's height, woman BMI as potential confounders and twins for clustering within a houseold. Not corrected for multiple outcomes or comparisons.
Group A: Preconception, pregnancy and early childhood intervention; **Group B**: Only preconception intervention; **Group C**: Only pregnancy and early childhood intervention; **Group D**: Control

Group A+B vs Group C+D: Effect of preconception interventions: women who received interventions compared with those who did not receive preconception interventions ; **Group A+C vs Group B+D**: Effect of pregnancy and early childhood interventions: women who received these interventions compared with those who did not receive these interventions ; **Group A vs Group D:** Effect of preconception, pregnancy and early childhood interventions: women who received interventions in these periods compared with those who received routine care.

Table S12. Secondary outcomes: Dietary intake data at infant ages 9, 12, 18 and 24 months

Outcomes	Groups				Preconception intervention groups vs no preconception intervention groups A+B vs C+D		Pregnancy intervention groups vs no pregnancy intervention group A+C vs B+D		Preconception and pregnancy intervention group vs no preconception and pregnancy intervention group A vs D	
	A	B	C	D	IRR (98.3%)	ARR (%) or MD (98.3%)	IRR (98.3%)	ARR (%) or MD (98.3%)	IRR (98.3%)	ARR (%) or MD (98.3%)
**Mean (SD) total calories consumed (kcal)***										
9 mo	n=65 565.2 (336.9)	n=52 396.1 (310.7)	n=45 605.7 (317.6)	n=49 406.1 (277.7)		-7.82 (-115.88 to 100.24)		172.85 (70.36 to 275.33)		144.79 (-4.97 to 294.54)
12 mo	n=62 777.8 (318.8)	n=70 437.4 (285.7)	n=45 718.9 (288.9)	n=48 565.3 (377.4)		-46.96 (-159.65 to 65.72)		258.89 (152.68 to 365.10)		218.69 (46.17 to 391.21)
18 mo	n=70 978.5 (383.9)	n=55 696.9 (340.7)	n=40 907.1 (322.9)	n=51 607.6 (306.0)		124.94 (5.52 to 244.36)		289.88 (173.85 to 405.92)		339.99 (190.05 to 489.93)
24 mo	n=116 1031.5 (382.0)	n=120 691.0 (299.9)	n=72 1039.2 (283.7)	n=68 772.6 (282.7)		-45.72 (-131.13 to 39.70)		312.03 (226.80 to 397.25)		249.50 (127.26 to 371.74)
**Mean (SD) total protein consumed (g)***										
9 mo	n=65 17.7 (11.3)	n=52 13.1 (10.8)	n=45 18.6 (12.5)	n=49 13.2 (9.4)		-0.09 (-3.88 to 3.70).		4.43 (0.78 to 8.07)		3.60 (-1.65 to 8.85)
12 mo	n=62 22.9 (11.2)	n=70 14.5 (10.8)	n=45 21.5 (9.1)	n=48 18.7 (14.1)		-1.78 (-5.70 to 2.14)		5.82 (1.98 to 9.66)		4.14 (-2.10 to 10.39)
18 mo	n=70 29.2 (11.3)	n=55 21.6 (12.0)	n=40 25.8 (9.7)	n=51 18.2 (11.7)		4.62 (0.76 to 8.47)		7.71 (3.71 to 11.70)		9.75 (4.46 to 15.04)
24 mo	n=116 29.7 (12.2)	120 20.2 (10.2)	72 30.0 (10.8)	n=68 22.4 (9.5)		-1.27 (-4.13 to 1.58)		8.89 (6.09 to 11.70)		6.89 (2.96 to 10.82)
**Received food from ≥5 food groups (minimum dietary diversity)†**										
12 mo - n/total (%)	778/949 (82.0)	654/919 (71.2)	582/718 (81.1)	499/683 (73.1)	0.99 (0.95 to 1.04)	-0.70 (-4.29, 2.89)	1.14 (1.09 to 1.20)	10.12 (6.39, 13.84)	1.13 (1.05 to 1.21)	9.49 (4.05, 14.93)
24 mo - n/total (%)	334/412 (81.1)	281/405 (69.4)	220/269 (81.8)	179/249 (71.9)	0.98 (0.91 to 1.05)	-1.81 (-7.61, 4.00)	1.16 (1.07 to 1.25)	10.85 (4.74, 16.97)	1.11 (0.99 to 1.24)	7.41 (-1.53, 16.35)

*Adjusted mean difference (98.3% CI), †Adjusted Incidence Rate Ratio (IRR), 98.3% CI, adjusted for place of birth, family possesses below poverty line card, woman's height, woman BMI as potential confounders and twins for clustering within a household. Not corrected for multiple outcomes or comparisons.

Group A: Preconception, pregnancy and early childhood intervention; **Group B**: Only preconception intervention; **Group C**: Only pregnancy and early childhood intervention; **Group D**: Control

Group A+B vs Group C+D: Effect of preconception interventions: women who received interventions compared with those who did not receive preconception interventions ; **Group A+C vs Group B+D**: Effect of pregnancy and early childhood interventions: women who received these interventions compared with those who did not receive these interventions ;

Group A vs Group D: Effect of preconception, pregnancy and early childhood interventions: women who received interventions in these periods compared with those who received routine care.

Table S13. Secondary outcomes: Morbidity at infant ages 1, 3, 6, 12, 18 and 24 months

Outcomes	Groups				Preconception intervention groups vs no preconception intervention groups A+B vs C+D		Pregnancy intervention groups vs no pregnancy intervention group A+C vs B+D		Preconception and pregnancy intervention group vs no preconception and pregnancy intervention group A vs D	
	A	B	C	D	IRR (98.3%)	ARR (%) or MD (98.3%)	IRR (98.3%)	ARR (%) or MD (98.3%)	IRR (98.3%)	ARR (%) or MD (98.3%)
**Illness in neonatal period**										
	**n=1266**	**n=1258**	**n=1080**	**n=1073**						
Possible serious bacterial infection[†]–n (%)	174 (13.7)	168 (13.4)	132 (12.2)	166 (15.5)	0.97 (0.81 to 1.16)	-0.38 (-2.81 to 2.05)	0.87 (0.72 to 1.05)	-1.84 (-4.42 to 0.74)	0.83 (0.64 to 1.07)	-2.81 (-6.64 to 1.02)
Local infection[‡]–n (%)	75 (5.9)	53 (4.2)	73 (6.8)	50 (4.7)	0.89 (0.66 to 1.19)	-0.58 (-2.17 to 1.01)	1.31 (0.97 to 1.78)	1.42 (-0.40 to 3.23)	1.11 (0.71 to 1.72)	0.52 (-1.94 to 2.98)
Admitted in hospital one or more times–n (%)	71 (5.6)	56 (4.5)	56 (5.2)	54 (5.0)	0.97 (0.71 to 1.32)	0.18 (-1.35 to 1.70)	0.95 (0.69 to 1.32)	-0.40 (-2.04 to 1.25)	0.91 (0.57 to 1.45)	-0.21 (-2.55 to 2.13)
**Illness in 2 week period preceding interview**										
**Diarrhea[§]**										
3 months, n/total (%)	74/1085 (6.8)	65/1035 (6.3)	55/909 (6.1)	52/890 (5.8)	1.11 (0.82 to 1.51)	0.70 (-1.16 to 2.57)	1.03 (0.75 to 1.42)	0.12 (-1.86 to 2.11)	1.16 (0.74 to 1.83)	0.78 (-2.00 to 3.56)
6 months, n/total (%)	107/1160 (9.2)	119/1099 (10.8)	86/953 (9.0)	83/915 (9.1)	1.10 (0.87 to 1.39)	0.86 (-1.39 to 3.12)	0.88 (0.69 to 1.13)	-1.31 (-3.70 to 1.09)	0.98 (0.68 to 1.40)	-0.15 (-3.54 to 3.24)
12 months, n/total (%)	78/953 (8.2)	97/921 (10.5)	62/722 (8.6)	43/687 (6.3)	1.27 (0.95 to 1.68)	1.86 (-0.47 to 4.18)	0.98 (0.73 to 1.31)	-0.14 (-2.60 to 2.32)	1.36 (0.87 to 2.15)	2.01 (-1.32 to 5.34)
18 months, n/total (%)	45/658 (6.8)	41/640 (6.4)	37/449 (8.2)	28/438 (6.4)	0.91 (0.62 to 1.33)	-0.68 (-3.35 to 1.99)	1.26 (0.84 to 1.89)	1.82 (-0.96 to 4.60)	1.20 (0.68 to 2.10)	1.63 (-2.09 to 5.36)
24 months, n/total (%)	18/453 (4.0)	23/439 (5.2)	14/293 (4.8)	11/271 (4.1)	1.02 (0.56 to 1.85)	0.48 (-2.24 to 3.19)	1.06 (0.57 to 1.99)	0.50 (-2.51 to 3.50)	1.19 (0.48 to 2.96)	0.54 (-3.47 to 4.56)
**Diarrhea with symptoms of dehydration or dysentery[ǁ]**										
3 months, n/total (%)	-	-	1/909 (0.1)	-	-	-	-	-	-	-
6 months, n/total (%)	1/1160 (0.1)	4/1099 (0.4)	4/953 (0.4)	3/915 (0.3)	0.65 (0.16 to 2.55)	-0.15 (-0.57 to 0.26)	0.58 (0.12 to 2.84)	-0.11 (-0.51 to 0.29)	0.20 (0.01 to 3.05)	-0.24 (-0.74 to 0.25)
12 months, n/total (%)	1/953 (0.1)	2/921 (0.2)	1/722 (0.1)	-	-	-	-	-	-	-
18 months, n/total (%)	1/658 (0.2)	-	-	1/438 (0.2)	0.71 (0.03 to 18.65)	-0.04 (-0.36 to 0.29)	1.33 (0.12 to 14.46)	-0.02 (-0.31 to 0.31)	1.00 (0.09 to 10.60)	-0.08 (-0.73 to 0.58)

Outcomes	Groups				Preconception intervention groups vs no preconception intervention groups A+B vs C+D		Pregnancy intervention groups vs no pregnancy intervention group A+C vs B+D		Preconception and pregnancy intervention group vs no preconception and pregnancy intervention group A vs D	
	A	B	C	D	IRR (98.3%)	ARR (%) or MD (98.3%)	IRR (98.3%)	ARR (%) or MD (98.3%)	IRR (98.3%)	ARR (%) or MD (98.3%)
24 months, n/total (%)	1/453 (0.2)	1/439 (0.2)	-	-	-	-	1.14 (0.09 to 15.00)	-0.00 (-0.47 to 0.46)	-	-
**Pneumonia¶**										
3 months, n/total (%)	3/1085 (0.3)	2/1035 (0.2)	1/909 (0.1)	-	-	-	2.26 (0.19 to 27.01)	0.10 (-0.20 to 0.39)	-	-
6 months, n/total (%)	2/1160 (0.2)	3/1099 (0.3)	2/953 (0.2)	3/915 (0.3)	0.88 (0.20 to 3.88)	-0.05 (-0.42 to 0.32)	0.48 (0.07 to 3.11)	-0.11 (-0.48 to 0.26)	0.57 (0.05 to 7.28)	-0.16 (-0.69 to 0.38)
12 months, n/total (%)	1/953 (0.1)	1/921 (0.1)	1/722 (0.1)	1/687 (0.2)	0.73 (0.06 to 8.28)	-0.04 (-0.33 to 0.26)	0.99 (0.06 to 15.48)	-0.00 (-0.30 to 0.29)	0.74 (0.07 to 8.14)	-0.04 (-0.47 to 0.39)
18 months, n/total (%)	-	-	-	-	-	-	-	-	-	-
24 months, n/total (%)	-	-	-	-	-	-	-	-	-	-
**Severe pneumonia****										
3 months, n/total (%)	-	-	1/909 (0.1)	-	-	-	-	-	-	-
6 months, n/total (%)	-	-	-	1/913 (0.1)	-	-	-	-	-	-
12 months, n/total (%)	-	-	-	-	-	-	-	-	-	-
18 months, n/total (%)	-	-	-	-	-	-	-	-	-	-
24 months, n/total (%)	-	-	-	-	-	-	-	-	-	-
**Admitted in hospital one or more times***										
1 to <6 months	79/1234 (6.4)	65/1215 (5.4)	65/1041 (6.2)	57/1025 (5.6)	0.99 (0.74 to 1.32)	0.17 (-1.51 to 1.85)	0.97 (0.72 to 1.33)	-0.21 (-1.98 to 1.55)	0.93 (0.59 to 1.46)	0.03 (-2.45 to 2.51)
≥6 to <12 months	3/1207 (0.3)	7/1187 (0.6)	4/1005 (0.4)	3/979 (0.3)	1.28 (0.41 to 4.00)	0.25 (-0.31 to 0.81)	0.56 (0.17 to 1.90)	-0.19 (-0.58 to 0.20)	0.61 (0.08 to 4.76)	-0.06 (-0.60 to 0.49)
≥12 to <18 months	-	1/979 (0.1)	1/757 (0.1)	-	0.71 (0.02 to 21.73)	-0.02 (-0.22 to 0.18)	1.06 (0.08 to 14.68)	-0.00 (-0.20 to 0.19)	-	-
≥18 to <24 months	1/715 (0.1)	1/704 (0.1)	-	-	-	--	-	-	-	-

*Adjusted Incidence Rate Ratio (IRR, 98.3% CI), adjusted for place of birth, family possesses below poverty line card, woman's height, woman BMI as potential confounder and twins for clustering within a household. Not corrected for multiple outcomes or comparisons; †Possible serious bacterial infection: Reported one of the

following – not able to breastfeed or feed, fever, cold to touch, lethargy or unconscious or convulsions or fast or difficult breathing or pneumonia (local term); ‡Local infection: Presence of skin infection or discharge from eyes or ears

§Diarrhea: Reported by mother or caregiver; ¶Pneumonia: History of cough reported by the mother or difficult breathing or chest indrawing; **Severe pneumonia: Pneumonia with one or more of the following danger signs – not able to breastfeed or feed, fever, cold to touch, lethargy or unconsciousness or convulsions or stridor

Symptoms of dehydration and dysentery: Not able to drink, lethargy or unconsciousness, restlessness or irritability and sunken eyes; dysentery: blood in stool

Group A: Preconception, pregnancy and early childhood intervention; **Group B**: Only preconception intervention; **Group C**: Only pregnancy and early childhood intervention; **Group D**: Control

Group A+B vs Group C+D: Effect of preconception interventions: women who received interventions compared with those who did not receive preconception interventions; **Group A+C vs Group B+D**: Effect of pregnancy and early childhood interventions: women who received these interventions compared with those who did not receive these interventions; **Group A vs Group D:** Effect of preconception, pregnancy and early childhood interventions: women who received interventions in these periods compared with those who received routine care

Table S14. Number of women who died in the preconception period

Outcomes	Comparison: Preconception interventions		IRR (98.3% CI)
	**Intervention (n=6722)**	**Control (n= 6778)**	
Deaths during preconception period, n (%)*	10 (0.2)	6 (0.1)	1.77 (0.51 to 6.12)

*Adjusted Incidence Rate Ratio (IRR, 98.3% CI) adjusted for family possesses below poverty line card, woman's height, woman BMI

Table S15. **Number of mothers and children who died during pregnancy or in the early childhood period**

**Outcomes**	**Groups**				**Preconception intervention groups vs no preconception intervention groups A+B vs C+D**	**Pregnancy intervention groups vs no pregnancy intervention group A+C vs B+D**	**Preconception and pregnancy intervention group vs no preconception and pregnancy intervention group A vs D**
	**A**	**B**	**C**	**D**	**IRR (98.3% CI)**		
	**n=1326**	**n=1326**	**n=1134**	**n=1135**			
Deaths during pregnancy, childbirth or up to 24 months after birth, n (%)*	6 (0.5)	8 (0.6)	2 (0.2)	5 (0.4)	1.72 (0.57 to 5.24)	0.64 (0.22 to 1.87)	1.14 (0.26 to 4.95)
**Live births**	**n=1290**	**n=1276**	**n=1093**	**n=1093**			
Deaths from birth to 28 days of age, n (%)†	20 (1.6)	21 (1.7)	9 (0.8)	27 (2.5)	0.97 (0.56 to 1.68)	0.52 (0.29 to 0.95)	0.66 (0.31 to 1.40)
Deaths from birth to 24 months of age, n (%)†	28 (2.2)	36 (2.8)	18 (1.6)	36 (3.2)	0.75 (0.52 to 1.08)	0.67 (0.45 to 1.00)	0.56 (0.33 to 0.95)

*Adjusted Incidence Rate Ratio (IRR, 98.3% CI), adjusted for family possesses below poverty line card, woman's height, woman BMI

†Adjusted Incidence Rate Ratio (IRR, 98.3% CI), adjusted for family possesses below poverty line card, woman's height, woman BMI as potential confounders and twins for clustering within a household

Group A: Preconception, pregnancy and early childhood intervention; **Group B**: Only preconception intervention; **Group C**: Only pregnancy and early childhood intervention; **Group D**: Control
Group A+B vs Group C+D: Effect of preconception interventions: women who received interventions compared with those who did not receive preconception interventions; **Group A+C vs Group B+D**: Effect of pregnancy and early childhood interventions: women who received these interventions compared with those who did not receive these interventions; **Group A vs Group D:** Effect of preconception, pregnancy and early childhood interventions: women who received interventions in these periods compared with those who received routine care
